# Mass spectrometric imaging and quantitative analysis of the *in vivo* biodistribution of trastuzumab using a rhodium(iii) sarcophagine complex

**DOI:** 10.1039/d5qi00731c

**Published:** 2025-08-26

**Authors:** Natasha Patel, Truc T. Pham, Arshiya Banu, Alex Griffiths, Brett M. Paterson, George Firth, Alexander Morrell, Clíona McMahon, Nicholas J. Long, James R. Baker, Vijay Chudasama, Michelle T. Ma

**Affiliations:** a School of Biomedical Engineering and Imaging Sciences, King's College London, St Thomas’ Hospital London UK michelle.ma@kcl.ac.uk; b London Metallomics Facility, King's College London Franklin Wilkins Building London UK; c Centre for Advanced Imaging, Australian Institute for Bioengineering and Nanotechnology, The University of Queensland Brisbane Australia; d Department of Chemistry, University College London Kathleen Lonsdale Building London UK; e Department of Chemistry, Imperial College London, Molecular Sciences Research Hub London UK

## Abstract

Mass cytometry with antibodies labelled with stable metal isotopes enables both sensitive imaging and the quantification of protein expression in biological samples. Typically, these specimens are exposed to a panel of labelled antibodies *ex vivo*, after sample collection. Here, we have developed a rhodium-labelled immunoconjugate of the HER2-targeted therapeutic IgG1 antibody, trastuzumab, and evaluated its *in vivo* biodistribution using mass cytometry techniques. A Rh^3+^ complex of a macrobicyclic sarcophagine (sar, 3,6,10,13,16,19-hexaazabicyclo[6.6.6]icosane) chelator was appended with a dibromopyridazinedione (DBPD), to produce a novel disulfide bond labelling molecule, “Rh-sar-DBPD”. Rh-sar-DBPD was site-specifically conjugated to trastuzumab *via* its four native solvent-accessible disulfide bonds, to yield a near homogeneous, well-defined and stable pyridazinedione (PD) immunoconjugate, Rh-sar-PD-trastuzumab, in which four Rh-sar-PD groups were attached per molecule of trastuzumab. Inductively coupled plasma mass spectrometry (ICP-MS) and laser ablation inductively coupled plasma mass spectrometry (LA-ICP-MS) were then applied to measure ^103^Rh content, as a proxy for Rh-sar-PD-trastuzumab accumulation, in *in vitro* and *in vivo* studies. ICP-MS *in vitro* studies indicated HER2-mediated uptake of Rh-sar-PD-trastuzumab in HER2-expressing breast cancer cells, with LA-ICP-MS images showing intercellular heterogeneity in Rh-sar-PD-trastuzumab uptake. To study the *in vivo* biodistribution of Rh-sar-PD-trastuzumab, female NSG mice bearing orthotopic HCC1954 breast cancer tumours were administered the immunoconjugate. Quantitative ICP-MS of ^103^Rh signal in dissected tissues indicated receptor-specific HER2-mediated uptake in tumours, as well as accumulation in the spleen and liver. Finally, LA-ICP-MS imaging analysis of tumour and ovary tissue sections showed heterogeneous uptake in HER2-expressing HCC1954 tumour cells and follicular granulosa cells of the ovaries, which are known to express growth factor receptors. To the best of our knowledge, this is the first report in which both ICP-MS and LA-ICP-MS have been used on tissue exposed to a metal-tagged antibody *in vivo*, enabling quantification of the biodistribution of the novel immunoconjugate, Rh-sar-PD-trastuzumab, in a murine model of breast cancer.

## Abstract 2

A new immunoconjugate, Rh-sar-PD-trastuzumab, enables *in vivo* mapping of antibody biodistribution, and tissue imaging of HER2 expression, using ICP-MS methods.

## Graphical abstract

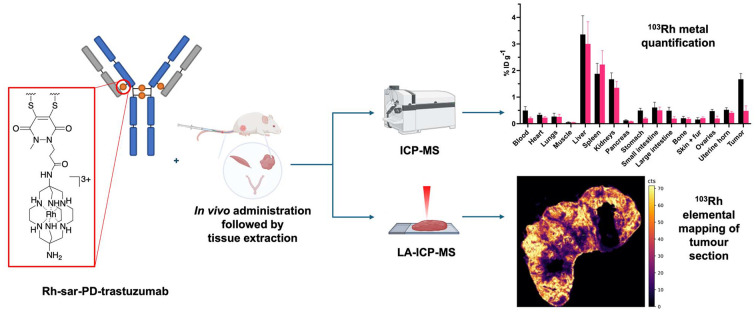

## Introduction

Inductively coupled plasma mass spectrometry (ICP-MS) and laser ablation inductively coupled plasma mass spectrometry (LA-ICP-MS) are widely employed to study the elemental distribution of metals in single cells, cell populations, animal models of disease and patient tissue samples. These technologies enable a variety of investigations including correlations between localisation of endogenous metals with biological function, insight into the accumulation of metallodrugs (*e.g.* cisplatin) and metal-based nanoparticles in tissues and organs, and mapping the biodistribution of biomolecules tagged with metal labels.^1^

Mass cytometry uses IgG antibodies tagged with monoisotopic, non-endogenous transition and lanthanide metal ions to provide information on protein expression. Biological samples are first exposed to a panel of antibodies, with each antibody tagged with a single stable isotope of a metal ion. Individual cells, populations of cells or tissue sections are then analysed by ICP-MS to measure metal content. The metal content serves as a metric for antibody accumulation, and therefore, protein expression. As mass spectrometry can quantitatively measure numerous metals in a single sample, a cocktail of antibodies, each with a unique metal tag, can provide a vast array of “multiplexed” information on protein expression and cell processes, from the single cell to the tissue level. Although novel and potentially useful complexes based on In,^2^ Te,^3^ Zr,^4^ Re and Pt^5^ have been explored, the most frequently employed commercially available mass cytometry tagging technologies^6–8^ use:

• polymers of DTPA (diethylenetriamine pentaacetic acid) and DOTA (1,4,7,10-tetraazacyclododecane-1,4,7,10-tetraacetic acid) chelators complexed to stable, single isotopes of metal ions, including lanthanide ions, and

• maleimide–thiol bioconjugation chemistry to attach the polymeric derivatives to reduced solvent-accessible disulfide bonds in an IgG. In IgG1 antibodies, eight reduced cysteine residues are available for attachment to these Ln-DOTA polymer tags, resulting in high degrees of metal loading or heterogeneous mixtures when the target has an average loading of less than eight.

**Fig. 1:**
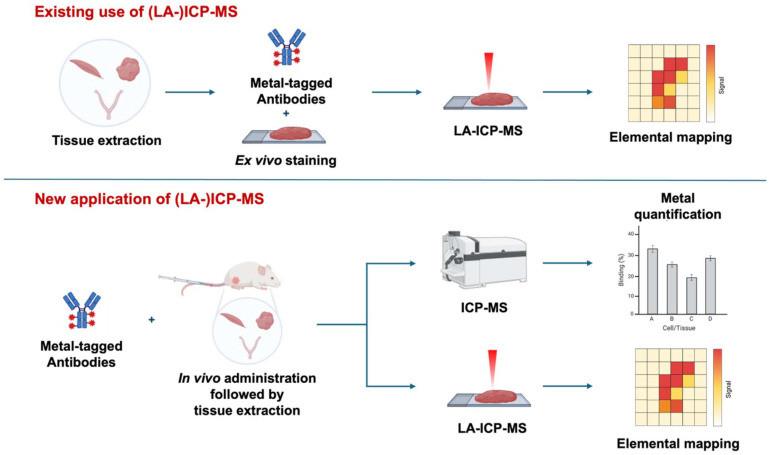
(a) Currently, panels of metal-labelled antibodies and LA-ICP-MS instrumentation are used to image protein expression of *ex vivo* tissue samples. (b) Here, we have designed a metal tag to stably label an antibody, and then administered the resulting immunoconjugate to mice intravenously. This chemical technology enables *in vivo* mapping of the immunoconjugate, and LA-ICP-MS images reflect the biodistribution/binding of the immunoconjugate to receptors in the *in vivo* environment.

In combination, LA-ICP-MS and mass cytometry antibodies have been used for *ex vivo* immunohistochemical profiling of protein expression^9–13^ (and other biomolecules, *e.g.* mRNA^14^) in tissue samples acquired from patients and animal models. Tissue sections are exposed to a panel of metal-tagged IgG antibodies (or other vectors) only *after* collection of samples from the *in vivo* environment (Fig. 1a). The information obtained is similar to that acquired using multiplexed fluorescently-tagged proteins for fluorescence microscopy studies. Critically, imaging mass cytometry enables greater multiplexing (≥40 channels) and lower background interference.^1^

Despite the adoption of mass cytometry and LA-ICP-MS to measure protein receptor expression, there have only been a limited number of ICP-MS and LA-ICP-MS imaging studies on the *in vivo* biodistribution of metal-tagged biomolecules (although not antibodies), in which the metal-tagged biomolecule is *first* administered to an animal, *prior* to tissue processing and LA-ICP-MS imaging analysis of tissue samples (Fig. 1b). Administration of a novel tellurium-tagged derivative of phenylalanine amino acid, TePhe, to a tumour-bearing mouse enabled highly resolved and specific LA-ICP-MS imaging of protein translation and synthesis pathways by mapping the distribution of ^130^Te in tissue sections.^15^ The biodistributions of a range of nanoparticles have also been studied *in vivo* using ICP-MS on tissue digests, *e.g.*, Pd-labelled PLGA drug delivery agents,^16^ Au dual diagnostic/therapeutic nanoparticles,^17,18^ and metal-labelled peptides and proteins.^19–22^

Our research aims to develop new chemical platforms that enable quantitative imaging and mapping of peptide and antibody biodistribution *in vivo* – to date, we have exclusively used radioactive metal ions for nuclear medicine applications.^23–27^ Other radiopharmaceutical scientists have substituted radioactive metal ions for stable isotopes in receptor-targeted peptides and proteins, and applied these isotopologues to interrogate the stability, biodistribution, metabolism and excretion of these bioconjugates *in vivo*.^21^ Understanding the pharmacokinetics of radiotracers is important to not only establish diagnostic and therapeutic efficacy, but also determine blood clearance times, excretion routes and radiation dose (and concomitant toxicity) to non-target tissues and organs.^21^

For example, ^90^Y-labelled β^−^-emitting antibodies were amongst the first receptor-targeted systemic radiotherapies to undergo clinical evaluation. To complement these innovations, antibody fragments were labelled with the stable isotope, ^89^Y, allowing ICP-MS quantification of ^89^Y excretion in urine.^22^ More recently, the radiotracers ^177^Lu-PSMA-617 and ^161^Tb-PSMA-617 have demonstrated efficacy in systemic radiotherapy of prostate cancer. ICP-MS in combination with the non-radioactive derivatives, ^175^Lu-PSMA-617 and ^159^Tb-PSMA-617, enabled quantification of the accumulation of these compounds in target prostate cancer tissue and non-target tissue, including excretory organs.^19^ Indeed, side-by-side γ-counting (using radioactive analogues) and ICP-MS studies yielded near-identical data. The *in vivo* plasma behaviours of disease-targeted peptides labelled with naturally abundant, non-radioactive isotopes of Ga and In (as proxies of radioactive ^68^Ga and ^111^In) have also been interrogated using ICP-MS. HPLC-ICP-MS studies of serum samples from rats administered these probes indicated distinct metabolic profiles of the Ga-labelled peptides compared to their In-labelled analogues, including transchelation of Ga^3+^ but not In^3+^ from the peptide to transferrin protein in blood plasma.^20^

Whilst these studies utilise ICP-MS only, the outcomes highlight that similar quantitative and qualitative ICP-MS information can be obtained using bioconjugates of non-radioactive metals in place of radioactive analogues. Here, we turn our attention to the use of a stable metal isotope, ^103^Rh, for tagging and mapping the biodistribution of an antibody at the whole body level, using not only ICP-MS, but LA-ICP-MS to provide high-resolution images of antibody accumulation in specific tissue. To the best of our knowledge, this is the first report in which mass cytometry imaging has been undertaken using tissue exposed to a metal-tagged antibody *in vivo*, and the first report in which the organ and tissue biodistribution of an antibody has been quantified using ICP-MS. We postulate that ICP-MS has applications in quantifying the biodistribution of new antibody-based drugs, revealing patterns of antibody accumulation in different tissue compartments, interrogating heterogeneity in antibody uptake and yielding valuable information on *in vivo* receptor expression.

## Results and discussion

### Selecting the antibody, metal complex and bioconjugation platform

Trastuzumab is a humanised IgG1 antibody that is routinely used for the treatment of HER2-positive breast cancer.^28^ Trastuzumab inhibits the proliferative effects of HER2 (human epidermal growth factor receptor 2) expression, which is over-expressed in 15–30% of breast cancers.^28^ Indeed, HER2 markers have been profiled in breast cancer mass cytometry studies.^9,10,13,29^ To both explore new chemical methodology for (LA-)ICP-MS and ensure the stability and integrity of the studied immunoconjugate *in vivo*, we have labelled trastuzumab, with the following functionalities:

(i) A Rh^3+^ complex of a (NH_2_)_2_sar chelator. Derivatives of a hexaazamacrobicyclic chelator, 3,6,10,13,16,19-hexaazabicyclo[6.6.6]icosane, commonly known as “sarcophagine” (sar), can chelate a range of metal ions,^30^ and are thus well suited to incorporating metal tags into proteins for the purposes of quantitative *in vivo* mapping and tissue imaging.^24,31^ Furthermore, low-spin d^6^ Rh^3+^, Ir^3+^ and Pt^4+^ ions form thermodynamically and kinetically stable complexes with sar, potentially expanding the mass cytometry chemical toolbox.^30,32^ Additionally, Rh possesses a single, stable naturally abundant isotope, ^103^Rh. Nature does not use Rh for biological functions; negligible amounts of endogenous ^103^Rh were expected in *in vitro* or *in vivo* studies described herein.

(ii) A dibromopyridazinedione (DBPD) functional group.^33–37^ The dibromopyridazinedione group reacts specifically with the thiols of a reduced disulfide bond, thus re-bridging the cysteine residues and enabling attachment of cargo to the biomolecule. Dithiol-substituted pyridazinedione (PD) bioconjugates, formed from the reaction of dibromopyridazinediones with reduced disulfides, are highly stable in blood serum, which is in contrast to the commonly-employed maleimide–thiol based bioconjugates, which are prone to retro-Michael reactions and ultimately the premature release of cargo or tags under *in vivo* conditions, compromising quantifiability of antibody distribution.^38–41^

### Synthesis and antibody conjugation

**Scheme 1:**
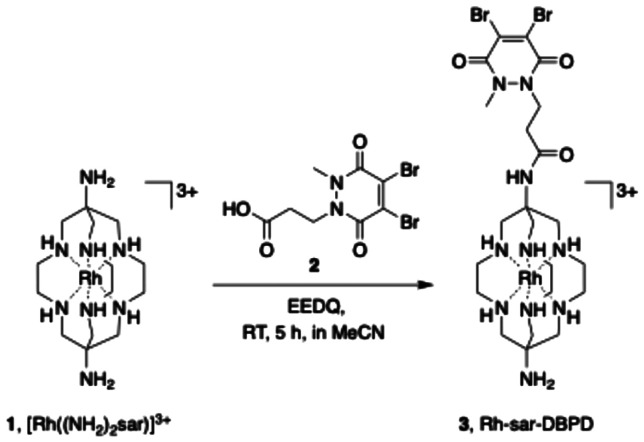
Amide coupling reaction of the sarcophagine complex [Rh((NH_2_)_2_sar)]^3+^ (1) with a dibromopyridazinedione acid derivative (2) to form Rh-sar-DBPD (3).

[Rh((NH_2_)_2_sar)](CF_3_SO_3_)_5_ (1) was reacted with dibromopyridazinedione acid derivative (2) and EEDQ (2-ethoxy-1-ethoxycarbonyl-1,2-dihydroquinoline), in an amide coupling reaction at ambient temperature, to produce Rh-sar-DBPD (3) (Scheme 1). Following reverse-phase HPLC purification, the new sarcophagine derivative was isolated in 12% yield, and characterised by NMR and HRMS (see Fig. S1–S3). We have previously reported similar low-yielding amide coupling reactions with [Mg((NH_2_)_2_sar)]^2+^, which we attributed to the lower reactivity of the apical primary amine groups of (NH_2_)_2_sar and its complexes relative to other primary amines, possibly due to steric encumbrance of the macrobicyclic hexaamine rings.^24^

**Scheme 2:**
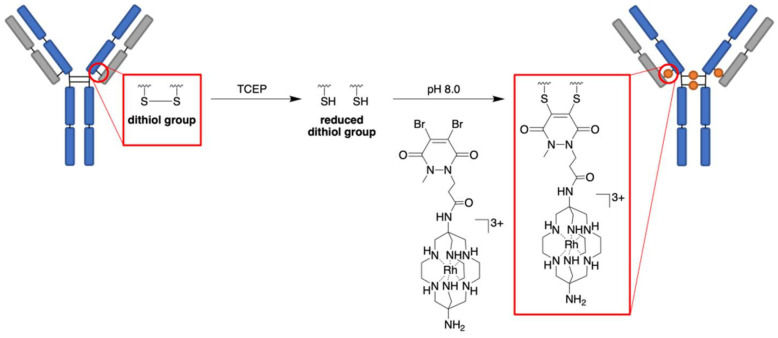
Antibody bioconjugation reaction in which the four interstrand disulfide bridges of trastuzumab IgG1 antibody are reduced, followed by re-bridging with Rh-sar-DBPD (3).

The reaction of Rh-sar-DBPD (3) with trastuzumab was optimized to achieve a payload to antibody (PAR) ratio of 4 (Table S1). The four solvent-accessible interstrand disulfide bonds of trastuzumab were reduced with mild reducing agent TCEP (tris(2-carboxyethyl)phosphine). Following removal of TCEP, Rh-sar-DBPD (10 equiv.) was reacted with the liberated cysteines (Scheme 2) by incubation in aqueous borate buffered saline (pH 8) at 37 °C for 3 h under constant agitation, to produce the desired pyridazinedione (PD) conjugate. Following buffer exchange into phosphate buffered saline (PBS) solution and purification using size-exclusion chromatography and spin filtration, the resulting Rh-sar-PD-trastuzumab conjugate was characterised by SDS-PAGE, UV-Vis spectroscopy and ESI-MS.

**Fig. 2:**
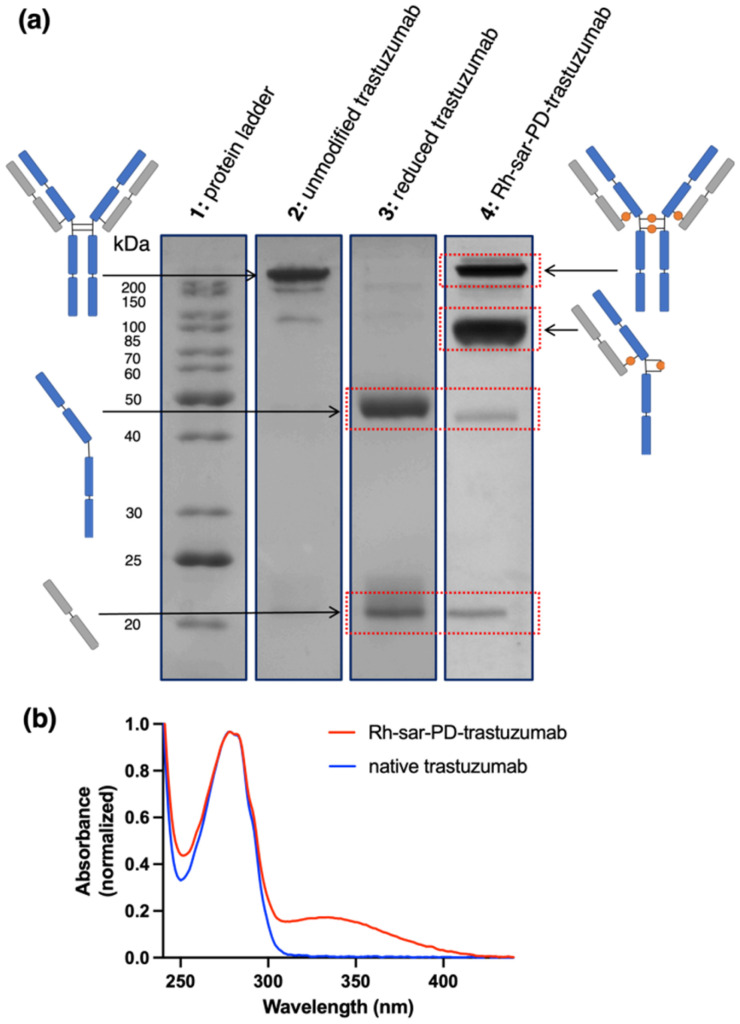
(a) SDS-PAGE of trastuzumab and the new immunoconjugate, Rh-sar-PD-trastuzumab; (b) UV-Vis spectra of Rh-sar-PD-trastuzumab, alongside native trastuzumab. Full gels are included in Fig. S5.

As expected, SDS-PAGE analysis of trastuzumab under TCEP-based reducing conditions showed the presence of separate heavy and light chains under the denaturing analysis conditions (Fig. 2a, lane 3) compared to the unmodified antibody (lane 2). For the new Rh-sar-PD-trastuzumab bioconjugate, the two major products observed by SDS-PAGE corresponded to the “full antibody” conjugate, in which the PD groups bridged the *inter*chain thiols at the hinge region, and the “half antibody” conjugate, in which the PD groups bridged *intra*chain thiols at the hinge region (Fig. 2a, lane 4). The full antibody re-bridging pattern resembles the native covalent linkages of unmodified trastuzumab. The half antibody fragment (comprising of a heavy chain and a light chain) arises because of non-native covalent linkages; this is commonly observed in dicysteine re-bridging reactions.^42,43^ Both re-bridging patterns result in an overall loading of four Rh-sar modules per full antibody. In physiological solutions (*e.g.* aqueous saline, PBS) and biological milieu (*e.g.* blood pool), two half antibody fragments are held together by non-covalent interactions in the Fc region. There is little to indicate that this “half antibody” product, containing *intra*chain-bridged thiols at the hinge region, would perform poorer *in vivo* with respect to HER2 and Fc receptor binding.^44^ Only very small amounts of heavy chain and light chain fragments were observed by SDS-PAGE; these were only visible when the SDS-PAGE gel was loaded with a highly concentrated sample. Our previous studies using dibromomaleimide derivatives of sarcophagine complexes achieved the same site-specificity, but led to higher observable amounts of heavy chain and light chain fragments in SDS-PAGE analyses,^24^ indicating the superior ability of the dibromopyridazinedione to produce minimal byproducts.

Dithiol-substituted pyridazinediones exhibit a distinct *λ*_max_ absorbance band at 335 nm (*ε*_335_ = 9100 M^−1^ cm^−1^), and the UV-Vis properties of trastuzumab are well-characterized (*ε*_280_ = 215 000 M^−1^ cm^−1^). UV-Vis spectroscopic analysis of the ratio of the 280 nm and 335 nm absorbance values was used to determine an average loading of 4.06 Rh-sar-PD linkages per antibody (Fig. 2b).

ESI-MS analysis was consistent with the SDS-PAGE and UV-Vis measurements. Deconvoluted ESI-MS spectra of the bioconjugate product revealed a relatively broad envelope of signals, corresponding to the presence of the “half antibody” species with the expected two Rh-sar-PD complexes per fragment, alongside the “full antibody” species with the expected four Rh-sar-PD complexes per fragment (Table 1 and Fig. S4). Experimental values fall with 12 a.m.u. of the calculated values. We also observed additional deconvoluted signals at *higher m*/*z* ratios for both half and full antibody [M]^+^ species (Fig. S4), leading to an envelope of multiple species. This “envelope” was significantly broader than that observed for unmodified trastuzumab and similarly prepared and characterized alkyne–trastuzumab immunoconjugates.^43,44^ We attribute this envelope to gas-phase adducts. For example, there is a strong “half-antibody” conjugate with a *m*/*z* signal of [M + 41]^+^, which we postulate is an acetonitrile adduct. We have previously observed this phenomenon with other sarcophagine derivatives.^24^

**Table 1:** ESI-MS data of Rh-sar-PD-trastuzumab

Half antibody conjugate	*m*/*z* (calculated)	*m*/*z* (observed)
Rh-sar-PD-trastuzumab		
Half antibody + 2 Rh-sar tags	73 775	73 782, 73 823 (+CH_3_CN adduct)
Full antibody + 4 Rh-sar tags	147 549	147 537

The ESI-MS signal corresponding to the “half antibody” had the highest intensity. The “full antibody” was observed at a much lower signal intensity. This is a common feature of such immunoconjugates because the smaller fragments are almost always detected with significantly higher relative intensity,^24,43^ likely due to mass spectrometric desolvation, ionisation and ion transport effects.^45^

The combination of SDS-PAGE, UV-Vis spectroscopic and ESI-MS analysis indicates the successful preparation of a highly homogeneous bioconjugate, in which each trastuzumab molecule contains an average of four Rh-sar-PD complexes site-specifically attached at each of the four native interchain disulfide groups of trastuzumab.

### *In vitro* uptake and imaging

**Fig. 3:**
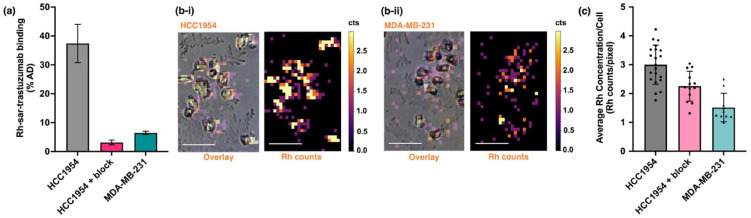
The ^103^Rh (LA-)ICP-MS data shows that Rh-sar-PD-trastuzumab uptake is significantly higher in HER2-positive HCC1954 human breast cancer cells when compared with HER2-negative MDA-MB-231 human breast cancer cells and HCC1954 cells co-administered with excess trastuzumab (HCC1954 + block). (a) *In vitro* binding of Rh-sar-PD-trastuzumab, based on ICP-MS quantification of ^103^Rh (data represented as percentage of the total added dose) (*n* = 4). (b) ^103^Rh LA-ICP-MS images of breast cancer cells after treatment with Rh-sar-PD-trastuzumab: (i) HER2-positive HCC1954 cells; (ii) HER2-negative MDA-MB-231 cells. Left: LA-ICP-MS image overlaid with bright field image; right: LA-ICP-MS image only. Scale bar corresponds to 100 μm. (c) Quantification of mean ^103^Rh counts per pixel in individual cells, measured using LA-ICP-MS.

Uptake of Rh-sar-PD-trastuzumab in the human breast cancer HCC1954 cell line, which exhibits high HER2 expression, was determined using ^103^Rh ICP-MS measurements. To assess specificity for the HER2 receptor, Rh-sar-PD-trastuzumab was incubated with: (i) HCC1954 cells; (ii) HCC1954 cells in the presence of a large excess of trastuzumab to block HER2 specific binding; and (iii) human breast cancer MDA-MB-231 cells, which have low HER2 expression (Fig. 3a). For cells incubated with Rh-sar-PD-trastuzumab, 37.4 ± 6.6% AD (percentage of the total added dose) of immunoconjugate was associated with HCC1954 cells. In HCC1954 cells co-incubated with a blocking dose of trastuzumab, this decreased to 3.1 ± 0.8% AD (mean difference = 34.3% AD, *p* = 0.002), and for MDA-MB-231 cells, this measured 6.5 ± 0.6% AD (mean difference = 30.9% AD, *p* = 0.003).

Preliminary LA-ICP-MS ^103^Rh imaging was undertaken using Rh-sar-PD-trastuzumab to assess the sensitivity of Rh-sar-PD-trastuzumab for detecting differential HER2 expression in single HER2-positive HCC1954 cells and HER2-negative MDA-MB-231 cells. In side-by-side experiments, the immunoconjugate was incubated with either HCC1954 cells (with and without excess trastuzumab as a blocking agent) or MDA-MB-231 cells. After washing and fixing, cells were imaged using a bright field light microscope and LA-ICP-MS for ^103^Rh signal at a resolution of 8 μm. The spatial distribution of the ^103^Rh signal corresponded with the breast cancer cells. Quantification of ^103^Rh LA-ICP-MS images indicated that a significantly higher average concentration of ^103^Rh was associated with HCC1954 cells compared with MDA-MB-231 cells (Fig. 3b). For HCC1954 cells, the average ^103^Rh concentration measured 3.00 ± 0.68 counts per pixel (*n* = 21); for MDA-MB-231 cells the average ^103^Rh concentration measured 1.51 ± 0.49 counts per pixel (*n* = 10, mean difference = 1.49, *p* < 0.0001) (Fig. 3c). Additionally, HCC1954 cells incubated with Rh-sar-PD-trastuzumab co-administered with a blocking dose of trastuzumab showed a decreased average ^103^Rh concentration (2.26 ± 0.52 counts per pixel, *n* = 13) compared with that of HCC1954 cells administered Rh-sar-PD-trastuzumab only (mean difference = 0.74, *p* = 0.005). Furthermore, in HCC1954 and MDA-MB-231 cells, there was a significant “spread” in the average ^103^Rh concentration measured for each cell. This is possibly due to intercellular heterogeneity in HER2 expression or intercellular heterogeneity in immunoconjugate binding.

Potassium signal, specifically ^39^K, is often used to delineate cells in LA-ICP-MS studies. In a separate experiment, the distributions of both ^103^Rh and ^39^K were simultaneously measured in HCC1954 cells treated with Rh-sar-PD-trastuzumab. As expected, the spatial distribution of ^39^K signal correlated with ^103^Rh signal (Fig. S6).

These *in vitro* studies indicated that (LA-)ICP-MS in combination with a new Rh-sar appended trastuzumab immunoconjugate can provide information on the *relative* expression of HER2 in both bulk tissue culture, and also at the single cell level, with data correlating with known HER2 expression levels in HCC1954 and MDA-MB-231 breast cancer cells.^46^ We note that analysis of a larger population of these cells would enable more robust quantification of antibody uptake as a proxy for HER2 expression. Similar data could also be obtained using single cell ICP-MS analyses.

### ICP-MS quantification of *in vivo* uptake

**Fig. 4:**
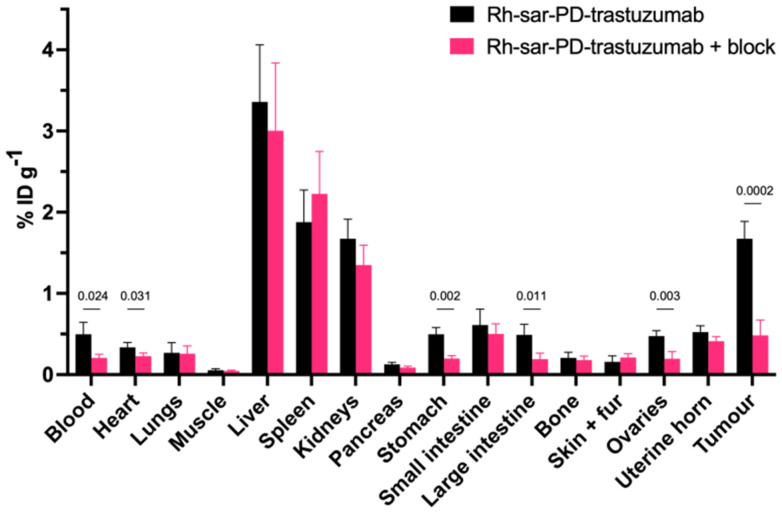
*Ex vivo* biodistribution of female NSG mice bearing orthotopic HCC1954 tumours (*n* = 4), administered either Rh-sar-PD-trastuzumab (500 μg) or Rh-sar-PD-trastuzumab (500 μg) with excess trastuzumab (1 mg) to block HER2 receptors. Quantification is based on ^103^Rh ICP-MS measurements of tissue digests. Error bars correspond to standard deviation, and where statistically significant blocking effects are observed, *p* values >0.05 are included. Data is also included in Table S6.

The biodistribution of the Rh-sar-PD-trastuzumab immunoconjugate was studied in a murine model of breast cancer (Fig. 4 and Table S6). Here, female NOD scid gamma (NSG) mice bearing orthotopic HCC1954 tumours were intravenously administered Rh-sar-PD-trastuzumab (500 μg). Mice were culled 72 h post-administration of Rh-sar-PD-trastuzumab, and organs were harvested and digested, followed by ^103^Rh ICP-MS analysis. The resulting data was processed to give a measurement of *percentage injected dose per gram of wet tissue* (%ID g^−1^) for each sample. To assess HER2-specificity of Rh-sar-PD-trastuzumab accumulation in tumours, a second group of mice was intravenously administered with unmodified trastuzumab (1 mg) to block HER2-mediated uptake of the immunoconjugate. Clinical and preclinical data has indicated that trastuzumab requires 2–5 days to clear circulation and accumulate at target tumour tissue. Therefore, this trastuzumab dose was administered 2 days prior to that of Rh-sar-PD-trastuzumab, to ensure adequate uptake/“blocking” of HER2 receptors.

The measured ^103^Rh content was 1.67 ± 0.21%ID g^−1^ in HER2-expressing tumour tissue (Fig. 4 and Table S6). The ^103^Rh content decreased in tumours of mice pre-administered with excess trastuzumab: in this “blocked” group, tumour ^103^Rh content measured 0.48 ± 0.19%ID g^−1^ (mean difference = 1.19 ± 0.21%ID g^−1^, *p* = 0.0002), consistent with trastuzumab blocking HER2-mediated uptake of Rh-sar-PD-trastuzumab immunoconjugate.

Statistically significant “blocking” effects were also observed in ovarian, heart, stomach and large intestine tissues, and blood (Fig. 4 and Table S6). We attributed some Rh-sar-PD-trastuzumab uptake, alongside trastuzumab “blocking” effects in heart, gastrointestinal and blood samples, to reactivity between the humanised Fc domain of Rh-sar-PD-trastuzumab/trastuzumab and murine Fc-receptors. The Fc domains of humanised IgG1 therapeutic antibodies have affinity for murine Fc-receptors, including (i) FcγR of effector immune cells such as macrophages and neutrophils that circulate in the blood and also reside in the spleen, and (ii) FcRn, expressed in the gastrointestinal epithelium, vascular endothelium, liver and immune cells.^47,48^ The immune deficient NSG mice used in this study lack B cells, and therefore also lack normal endogenous circulating murine immunoglobulins that would normally compete with trastuzumab/Rh-sar-PD-trastuzumab for Fc-receptors in immunocompetent mice. It is likely that the pre-administered dose of excess, competing trastuzumab reduces binding of the Fc domain of Rh-sar-PD-trastuzumab to murine FcγR and FcRn, leading to lower concentrations in compartments known to contain murine FcγR and FcRn Fc-receptors, such as the blood pool and gastrointestinal tract.

For both groups of mice, there were also significant amounts of ^103^Rh measured in organs in which IgG antibodies are known to residualise, specifically the spleen and liver. Effector immune cells, which express Fc-receptors, are also well-documented to reside in the spleen and liver, and the observed Rh-sar-PD-trastuzumab biodistribution patterns are consistent with prior studies reporting radiolabelled trastuzumab in mouse models.^24,25,49^

Administration of trastuzumab (1 mg) 2 days prior to Rh-sar-PD-trastuzumab (500 μg) resulted in decreased ^103^Rh content in ovarian tissue, from 0.48 ± 0.07%ID g^−1^ to 0.20 ± 0.09%ID g^−1^ (mean difference = 0.28 ± 0.21%ID g^−1^, *p* = 0.003). This suggests that Rh-sar-PD-trastuzumab uptake in ovarian tissue is mediated by receptor-specific interactions. We note that human ovarian and gastrointestinal tract tissue are known to express HER2,^50^ and in mice, the equivalent *neu* receptor is also expressed at low levels in these organs.^51,52^ However, trastuzumab does not exhibit affinity/cross-reactivity for murine *neu* receptors.^53^ It is therefore unlikely that the blocking effects observed in healthy murine tissue are a result of interactions between the Fv domain of trastuzumab/Rh-sar-PD-trastuzumab and murine *neu* receptor. We postulate that ^103^Rh accumulation in ovarian tissue is due to interactions between trastuzumab/Rh-sar-PD-trastuzumab and other murine growth factor receptors (*vide infra*).

### LA-ICP-MS imaging of tumour and ovarian tissue

**Fig. 5:**
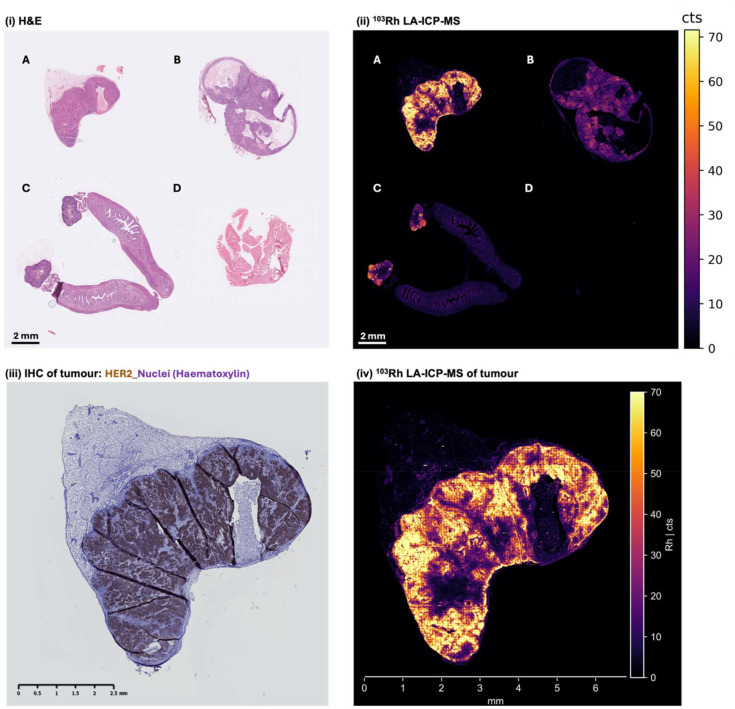
(i) Haematoxylin and eosin image and (ii) ^103^Rh LA-ICP-MS image (resolution 10 μm) of formalin-fixed, paraffin-embedded sections of: (A) tumour from a mouse administered Rh-sar-PD-trastuzumab; (B) tumour from a mouse administered trastuzumab, followed by Rh-sar-PD-trastuzumab; (C) ovaries and uterine horn from a mouse administered Rh-sar-PD-trastuzumab; (D) muscle from a mouse administered Rh-sar-PD-trastuzumab. Contiguous sections of the tumour from (A), showing similar heterogeneous expression of HER2, as measured by (iii) conventional immunohistochemistry (HER2: brown stain; cell nuclei: haematoxylin blue stain) and (iv) ^103^Rh LA-ICP-MS.

Tumour, ovaries and uterine horn and muscle samples from the above groups of mice were collected, paraffin-embedded and sectioned at 10 μm for *ex vivo* tissue analysis. Selected sections were analysed by LA-ICP-MS for ^103^Rh signal at a resolution of 10 μm, with contiguous sections then undergoing haematoxylin and eosin (H&E) staining in order to identify structures and cell types (Fig. 5).

For mice treated with either Rh-sar-PD-trastuzumab or Rh-sar-PD-trastuzumab + blocking trastuzumab, LA-ICP-MS imaging of tumour sections revealed ^103^Rh signal in tumour tissue, but negligible signal in surrounding adipose or necrotic/apoptotic/acellular tissue. Consistent with ICP-MS biodistribution data, ^103^Rh signal for the tumour section from the group administered Rh-sar-PD-trastuzumab showed higher ^103^Rh signal intensity compared to the group co-administered a blocking dose of trastuzumab. These data are consistent with high HER2 specificity of the Rh-sar-PD-trastuzumab immunoconjugate.

Additionally, ^103^Rh signal in tumour tissue was heterogeneous. Quantification of selected areas in these tumour sections demonstrated this. For a tumour section of a mouse administered Rh-sar-PD-trastuzumab only, ^103^Rh signal measured up to 100 counts per pixel in one area of tumour section, but more typically between 40–46 counts per pixel in other delineated areas (Fig. S7). Although lower ^103^Rh values were observed for a tumour section of a mouse administered Rh-sar-PD-trastuzumab + blocking trastuzumab, ^103^Rh signal was similarly heterogeneous, with uptake measuring up to 30 counts per pixel in one area of tumour, but as low as 9 counts per pixel in an alternately delineated area (Fig. S8).

To validate HER2 expression patterns in the tumour correlated with ^103^Rh signal, immunohistochemistry (IHC) analysis was undertaken on a contiguous tumour section from a mouse treated with Rh-sar-PD-trastuzumab. Here, an anti-HER2 primary antibody that binds to a HER2 epitope distinct from that of trastuzumab was used to treat tumour sections,^54^ prior to staining protocols (with a secondary antibody) including counterstaining with haematoxylin and finally, imaging. IHC (Fig. 5) shows that (i) orthotopic HCC1954 tumours indeed express HER2, (ii) HER2 expression is heterogeneous and (ii) in contiguous sections, HER2 expression, as measured by IHC, spatially correlates with ^103^Rh signal, as measured by LA-ICP-MS using Rh-sar-PD-trastuzumab.

Monoclonal therapeutic IgG antibodies, including trastuzumab, are known to exhibit heterogeneous intratumoral and intertumoral uptake in diseased tissue, either as a result of poor tissue perfusion or heterogeneous target receptor expression.^55–57^ In breast cancer, this heterogeneity has been associated with resistance to antibody therapies.^28^ The ability to quantify and image *in vivo* heterogeneous distribution of a therapeutic antibody using elemental mass spectrometry potentially enables insight into the cause of differences in immunotherapy outcomes.

Sections of muscle, which do not express HER2 or its murine-equivalent, the neu receptor, showed negligible Rh-sar-PD-trastuzumab uptake, with a maximum ^103^Rh intensity of 1 count.

LA-ICP-MS images of ovarian sections revealed an interesting distribution of ^103^Rh signal, prompting us to closely compare ^103^Rh LA-ICP-MS images with ovarian structures in H&E images. Classification of ovarian follicles is complex.^58–61^ We therefore confined ourselves to basic classifications of the mouse ovary^58,59^ – that is, identifying follicle structures, oocytes, granulosa cells of follicles, corpus luteum and vasculature.

**Fig. 6:**
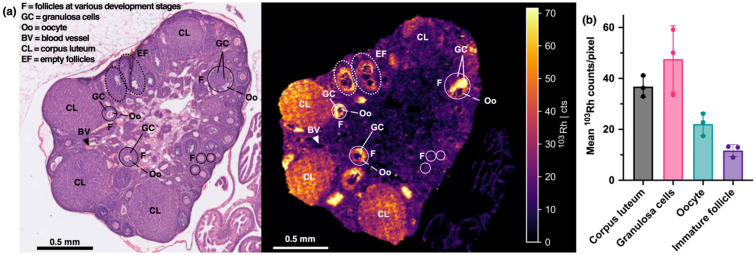
(a) Haematoxylin and eosin image (left) and ^103^Rh LA-ICP-MS image (resolution 10 μm) (right) of a section of a formalin-fixed, paraffin-embedded ovary from a mouse treated with Rh-sar-PD-trastuzumab. (b) Quantification of ^103^Rh signal in ovarian structures.

^103^Rh signal was coincident with ovarian granulosa cells (GC), which are contained within developing ovarian follicles (F) and surround the oocyte (Oo, colloquially known as the “egg”) (Fig. 6). Importantly, granulosa cells express growth factors and growth factor receptors (including human HER2/murine *neu* receptors, amongst other members of the epidermal growth factor receptor family).^51,62–64^ During follicle development and maturation, granulosa cells surrounding the oocyte expand, and the interplay of sex hormones with growth factors and growth factor receptors stimulates oocyte development prior to ovulation. After ovulation, where the oocyte is released from the ovary, the corpus luteum (CL) develops from the remains of the follicle. The corpus luteum produces hormones to support pregnancy; in the absence of pregnancy, the corpus luteum decays.

In H&E images of ovarian sections, follicles at various development stages were identified, including immature follicles without granulosa cells, maturing follicles with significant granulosa cell numbers, and empty follicles (EF). Corpus luteum structures were also observed. Quantification of LA-ICP-MS images (Fig. 6 and S9) revealed the presence of ^103^Rh signal/Rh-sar-PD-trastuzumab accumulation in granulosa cells (33–59 counts per pixel) and corpus luteum structures (33–41 counts per pixel) but lower amounts in oocytes (17–26 counts per pixel) and immature follicles (9–13 counts per pixel).

Although it is unlikely that Rh-sar-PD-trastuzumab binds to *neu* receptors expressed in murine granulosa cells,^53^ we postulate that there is some cross-reactivity between Rh-sar-PD-trastuzumab and other murine growth factor receptors of granulosa cells. This is consistent with reports that murine granulosa cells (including differentiated granulosa cells in mature follicles), but not oocytes, express growth factor receptors.^51^ It is also possible that ^103^Rh signal is a result of Rh-sar-PD-trastuzumab binding to Fc-receptors of granulosa cells, however there is little known about Fc-receptor expression in follicle development.

These studies highlight the utility of LA-ICP-MS imaging in revealing the off-target accumulation of antibody therapies, and potential cross-reactivity between antibody therapies and non-target receptors, such as growth factor receptors in reproductive organs. Such insights are critical in understanding the safety profile of antibody therapies.

LA-ICP-MS images were also acquired of the uterine horn. Whilst the intensity of ^103^Rh signal in sections of the uterine horn was significantly lower than that of granulosa cells of ovarian follicles, there was still some appreciable signal, consistent with both ICP-MS data, and prior studies describing FcRn expression in uterine tissue, predominantly on epithelial tissue.^65^

## Concluding remarks

We have developed a new chemical mass cytometry platform and applied it to quantify and image the *in vivo* biodistribution of a novel trastuzumab immunoconjugate, Rh-sar-PD-trastuzumab. To the best of our knowledge, this is the first report describing quantitative ICP-MS biodistribution studies and LA-ICP-MS imaging of tissues collected from animals intravenously administered an immunoconjugate labelled with a stable metal isotope. Many studies have collected tissue, either *post-mortem* from animals, or from human patients, and only then exposed these samples to panels of antibodies for subsequent mass cytometry or LA-ICP-MS imaging, typically to assess antigen expression. Here, we have demonstrated that complementary ICP-MS and LA-ICP-MS analyses are sufficiently sensitive to enable interrogation of the behaviour of a therapeutic antibody at the whole-body *and* tissue level.

At the initiation of this project, we specified several criteria of a desired immunoconjugate, to enable accurate and reliable mass spectrometric analyses:

(i) First, the immunoconjugate needed to be highly stable *in vivo* so that quantification and imaging of ^103^Rh signal reflected the distribution of the trastuzumab immunoconjugate, rather than dissociated metal ion or metal complex. The combination of sarcophagine chemistry and dibromopyridazinedione chemistry ensured the formation of a highly stable immunoconjugate.

(ii) Second, the trastuzumab bioconjugation reaction needed to provide a highly homogeneous product, with reproducible site-specific attachment of cargo to the antibody. Immunoconjugates consisting of heterogeneous product mixtures often demonstrate reduced affinity for target receptors or undesirable biodistribution properties.^49,66,67^ In this study, SDS-PAGE, UV-Vis spectroscopy and ESI-MS analyses demonstrated that Rh-sar-PD-trastuzumab consisted of a homogeneous product, in which four Rh-sar-PD motifs site-specifically bridged at the native interstrand disulfide bonds of the antibody.

(iii) Third, it was highly desired that our trastuzumab immunoconjugate closely mimicked the unmodified trastuzumab, so that biological results from mass spectrometry analyses were relevant in the context of trastuzumab therapies. Existing mass cytometry immunoconjugates contain polymers of DOTA or DTPA complexes: whilst these polymers maximise mass spectrometric signal, it is possible that *in vivo*, the presence of multiple chains of metal complex polymers would influence the biodistribution. Here, only four Rh-sar motifs were attached per molecule of trastuzumab antibody. This provided sufficient sensitivity to enable not only ICP-MS quantification and LA-ICP-MS imaging of Rh-sar-PD-trastuzumab in tumours, but also mapping of Rh-sar-PD-trastuzumab accumulation in other tissues, providing information on the biodistribution of trastuzumab itself *in vivo*. Additionally, having a defined payload of four ^103^Rh atoms per antibody, rather than a polymer with variability in payload number, could enable more accurate quantification of antibody accumulation in tissue and/or receptor expression in tissue.

Currently, whole body quantification of antibody biodistribution can be undertaken with radioactive isotopes and either PET or SPECT imaging (depending on the radioisotope), and/or radioactivity counting of organs *ex vivo*.^23–25,49^ Molecular imaging of tissue or cellular structures using antibody derivatives is typically achieved using optical methods, including fluorescence imaging. Here, we have demonstrated that immunoconjugates such as Rh-sar-PD-trastuzumab are well-poised to enable both quantitative biodistribution and highly resolved molecular imaging at the tissue and cellular level. LA-ICP-MS enabled insight into the heterogeneous breast cancer cell uptake of Rh-sar-PD-trastuzumab *in vitro* and uptake in HER2-positive HCC1954 breast cancer tumours, and for the first time, in developing ovarian follicles *in vivo*.

Immunoconjugates containing stable metal isotope labels have had extraordinary impact in ICP-MS and LA-ICP-MS mass cytometry platform technologies. We postulate that the immunoconjugates disclosed in this manuscript, as well as derivatives thereof, could have further utility in an *in vivo* setting, which could lead to new applications in the biomedical research realm, and enable *in vivo* multiplexing for both ICP-MS biodistribution and LA-ICP-MS imaging analyses.

## Author contributions

NP designed experiments, undertook experimental work, collated data and drafted the manuscript, MTM conceived research, designed experiments, collated data and drafted the manuscript, TTP designed and undertook animal research, AB advised on, undertook and collated IHC research, GF undertook animal research, AG and AM advised on, undertook and collated ICP-MS and LA-ICP-MS research, CM advised on and undertook antibody conjugate analysis (SDS-PAGE, UV-Vis, MS analysis), BMP, NL, VC and JRB contributed to experimental design and advised on conception of research. All authors contributed to review and interpretation experimental data and editing the manuscript.

## Conflicts of interest

There are no conflicts to declare.

## Supplementary Material

QI-012-D5QI00731C-s001

## Data Availability

Access to further data (*e.g.* mass spectrometric imaging data) can be provided by emailing the corresponding author. Supplementary information: details on experimental methods, characterisation data and techniques (NMR, MS, (LA-)ICP-MS) and additional images and spectra. See DOI: https://doi.org/10.1039/d5qi00731c.
