# Supplementary material for: Mass spectrometric imaging and quantitative analysis of the in vivo biodistribution of trastuzumab using a rhodium(iii) sarcophagine complex
Source: Inorg Chem Front. 2025 Aug 26;12(19):5688–700. doi: 10.1039/d5qi00731c (PMC12400293; doi:10.1039/d5qi00731c)
Supplement: QI-012-D5QI00731C-s001 [file QI-012-D5QI00731C-s001.pdf]

Supplementary Information (SI) for Inorganic Chemistry Frontiers.
This journal is © the Partner Organisations 2025

**Mass spectrometric imaging and quantitative analysis of the *in vivo* biodistribution of trastuzumab using a rhodium(III) sarcophagine complex**

***Supporting Information***

*Natasha Patel[1], Truc T. Pham[1], Arshiya Banu,[1] Alexander Griffiths[2], Brett M. Paterson[3], George Firth[1], Alexander Morrell[2], Clíona McMahon[4], Nicholas J. Long[5], James R. Baker[4], Vijay Chudasama[4], Michelle T. Ma*[1]*

*[1]School of Biomedical Engineering and Imaging Sciences, King's College London, St Thomas' Hospital, London, United Kingdom*

*[2]London Metallomics Facility, King's College London, Franklin Wilkins Building, London, United Kingdom*

*[3]Centre for Advanced Imaging, Australian Institute for Bioengineering and Nanotechnology, The University of Queensland, Brisbane, QLD 4072, Australia.*

*[4]Department of Chemistry, University College London, Kathleen Lonsdale Building, London, United Kingdom*

*[5]Department of Chemistry, Imperial College London, Molecular Sciences Research Hub, London, United Kingdom*

*Corresponding author: Michelle Ma, King's College London, michelle.ma@kcl.ac.uk

**Table of Contents** **Page**

**Experimental** 2

**Figure S1.** $^{1}$H NMR spectra of Rh-sar-DBPD 9

**Figure S2.** $^{13}$C NMR spectra of Rh-sar-DBPD 9

**Figure S3.** HR-ESI-MS of Rh-sar-DBPD 10

**Figure S4.** Deconvoluted HR-ESI-MS spectra of trastuzumab derivatives 11

**Figure S5.** Full SDS-PAGE images of trastuzumab derivatives 12

**Figure S6.** $^{39}$K and $^{103}$Rh LA-ICP-MS imaging of breast cancer cells exposed to Rh-sar-PD-trastuzumab 12

**Figure S7.** $^{103}$Rh LA-ICP-MS tumour image quantification ($^{103}$Rh-sar-PD-trastuzumab) 13

**Figure S8.** $^{103}$Rh LA-ICP-MS tumour image quantification ($^{103}$Rh-sar-PD-trastuzumab + trastuzumab) 14

**Figure S9.** $^{103}$Rh LA-ICP-MS ovary image quantification ($^{103}$Rh-sar-PD-trastuzumab) 15

**Figure S10.** HER2 immunohistochemistry staining 16

**Table S1.** $\lambda_{max}$ values for Rh-sar-PD-trastuzumab and Co-sar-PD-trastuzumab 4

**Table S2.** LC-MS mobile phase gradient elution for trastuzumab derivative analysis 5

**Table S3.** Comparison of experimental conditions for reaction of Rh-sar-DBPD with trastuzumab 17

**Table S4.** Experimental data acquisition parameters used for ICP-MS 17

**Table S5.** Experimental data acquisition parameters used for LA-ICP-MS imaging 18

**Table S6.** Ex vivo biodistribution of Rh-sar-PD-trastuzumab based on $^{103}$Rh ICP-MS measurements of tissue digests 19

**References** 19

## Experimental

### *Materials and methods*

All solvents and reagents were purchased from commercial sources (Merck, Fischer Scientific, Fluorochem) and used without further purification, unless otherwise stated. MilliQ water (resistivity: 18.2 MΩ) was used to prepare all aqueous solutions. Semi-preparative HPLC purification was performed on an Agilent Prostar system using a Zorbax Eclipse C18 column (21.2 x 250 mm, 5 μm). $^1H$, $^{13}C\{^1H\}$, HSQC NMR data were acquired on Bruker 400 or 700 MHz machines with data being processed using MestReNova version 14.2.1 (700 MHz machine equipped with an AVIII console and a quadruple-resonance QCI cryoprobe). NMR spectra were referenced to solvent residual peaks, except in the case of $^{13}C$ spectra acquired in $D_2O$, which were referenced to residual trifluoroacetic acid.

ICP measurements were acquired on a Perkin Elmer NexION 350D Inductively Coupled Plasma Quadrupole Mass Spectrometer (ICP-QMS) under Kinetic Energy Discriminator (KED) mode at the London Metallomics Facility, King's College London. The introduction system to the instrument was a Cetac ASX-100 autosampler coupled to a SeaSpray glass nebuliser fitted to a quartz cyclonic spray chamber. Argon plasma flow and nebuliser gas flow rates were 18 L $min^{-1}$ and 0.98 L $min^{-1}$, respectively. Quality control of ICP-QMS measurements was ensured through repeat measurements of acid blanks, a calibrant and a certified reference material from High Purity Standards (CRM-TMDW-100). Analyte measurements were normalised to the internal Ga standard to account for instrument drift and matrix effects, and measurements were subsequently blank corrected by removing the average analyte intensity of repeat blank measurements. The corrected isotopic intensity was converted to concentration measurements by interpolation of neighbouring calibrants. The quality of the interpolation was confirmed by verifying the linearity of the calibration curve. Calibration standards were made volumetrically using a 100 mg $L^{-1}$ Sigma Aldrich ICP-MS Multi-element (VI) standard. A 0.8 M $HNO_3$ stock solution stock solution was made from Optima grade concentrated $HNO_3$ (68% w/w; Fisher Scientific trace metal grade acids) and purified water with a resistivity ≥18.2 MΩ cm from a Milli-Q system (Merck Millipore). This solution was also used to dilute the samples to ensure calibrants and samples were matrix matched. The concentrations of the calibrants were between 0.01 and 100 μg/L. All calibration solutions and blanks were doped with Leehman Labs (Teledyne) 100 mg/L Ga standard solutions to obtain a consistent concentration of 50 μg/L for all calibrants and samples as internal standard.

Statistical analysis was conducted using Prism 9.5.0 (GraphPad Software). Data are presented as mean ± SD. Statistical significance was determined using either Welch's t-tests or 1-way ANOVA followed by t-tests with multiple comparison correction (Tukey method).

### *Chemical synthesis*

*$[Rh(NH_3)_2sar](CF_3SO_3)_5$ (**1**):* $[Rh(NH_3)_2sar]Cl_5$ was synthesised from $[Rh(en)_3]Cl_3$ according to existing synthetic procedures.[1,2] Samples of $[Rh(NH_3)_2sar](CF_3SO_3)_5$ were then prepared by dissolving the chloride salts in trifluoromethanesulfonic acid, stirring the resulting solutions at room temperature for 30 min, and then precipitating the desired trifluoromethanesulfonate salt with diethyl ether. The $[Rh(NH_3)_2sar](CF_3SO_3)_5$ (**1**) precipitate was collected by filtration and washed with diethyl ether.

$[Rh(NH_3)_2sar](CF_3SO_3)_5$: $^1H$ NMR (400 MHz, $D_2O$) δ 3.75-3.61 (m, 12H, cage H), 3.01-2.91 (m, 12H, cage H).

*4,5-dibromo-2-methyl-3,6-dioxo-3,6-dihydropyridazinyl)propanoic acid (**2**)* was prepared according to existing synthetic protocols.[3] $^1H$ NMR (400 MHz, MeOD) δ 4.44 (t, *J* = 7.2 Hz, 2H), 3.69 (s, 3H), 2.74 (t, *J* = 7.3 Hz, 2H). $^{13}C$ NMR (101 MHz, MeOD) δ 173.7, 154.7, 154.4, 136.7, 136.3, 44.8, 35.4, 32.5.

*Rh-sar-DPD (**3**):* $[Rh(NH_3)_2sar](CF_3SO_3)_5$ (**1**), dibromopyridazinedione derivative (**2**), and EEDQ (2-ethoxy-1-ethoxycarbonyl-1,2-dihydroquinoline) were each dried under vacuum. Compound **2** and EEDQ were then added to dry acetonitrile (20 mL) and stirred for 1 h at ambient temperature. **1** was added to the reaction mixture, which was subsequently stirred for 4 h at ambient temperature. The reaction was quenched with glacial acetic acid (12.5 mL), and the solution concentrated under reduced pressure. Water (10 mL) was added to the oil. The supernatant was separated from insoluble species by centrifugation (3600 rpm, 15 min), decanted and then lyophilised. The resulting lyophilised material was redissolved in water and purified by reversed-phase preparative HPLC (Agilent PrepHT XDB-C18) using mobile phase A (water with 0.1 % trifluoroacetic acid) and mobile phase B (acetonitrile with 0.1 % trifluoroacetic acid) with an eluting gradient

(10 mL/min: 0-5 min 0 % B in A; 5-10 min 0 % → 20 % B in A; 10-40 min 20 % → 70 % B in A). The product was eluted in a single fraction (retention time 22.5 min) which was lyophilised.

Rh-sar-DBPD (**3**): $^1$H NMR (700 MHz, $D_2O$) δ 4.57-4.46 (m, 2H), 3.69–3.56 (m, 15H), 3.12–2.75 (m, 12H), 2.64-2.58 (m, 2H). $^{13}$C NMR (175 MHz, $D_2O$ referenced to TFA) δ 175.4, 157.4, 157.3, 138.5, 137.9, 60.9, 58.7, 57.0, 55.7, 54.1, 47.1, 38.0, 37.1. High resolution ESI-MS +ve mode, Rh-sar-DBPD (M = $[RhC_{22}H_{40}Br_2N_{10}O_3]^{3+}$). $[M - H]^{2+}$: observed = 377.0321, predicted = 377.0299.

See Figures S1 – S3.

*Antibody bioconjugation*

The method used was modified from reported literature.[4] Trastuzumab (5 mg) was obtained as Herzuma (biosimilar) in saline solution (19.6 mg/mL) from the Cancer Centre at Guy's Hospital, London. The solution was buffered exchanged into borate buffered saline (BBS; 25 mM sodium borate, 25 mM NaCl, 2 mM EDTA; pH 8.5) solution *via* spin filtration using a centrifugal filter (Vivaspin, 10 kDa MWCO). The concentration of trastuzumab was then adjusted to 2.91 mg/mL (1.37 mL).

Tris(2-carboxyethyl)phosphine solution (TCEP, 10 mM in BBS, 13.7 µL, 6 equiv.) was then added, and the solution was incubated at 37 °C for 1.5 h. The TCEP was removed *via* spin filtration (Vivaspin, 10 kDa MWCO), and the resulting solution containing antibody was diluted with BBS to 1.37 mL. Rh-sar-DBPD (**3**) (10 eq. in 13.7 µL of $H_2O$) was added and the reaction incubated at 37 °C for 3 h under constant agitation. The resulting Rh-sar-PD-trastuzumab bioconjugate was purified using PD-10 desalting (size exclusion) columns (Cytiva, UK). PBS was used as the solvent, and 500 µL fractions were collected. The protein concentration in each fraction was measured using UV spectroscopy (280 nm absorbance). Fractions containing the highest concentration of antibody conjugate were combined, and further purified (in PBS) by six cycles of spin filtration using a centrifugal filter (Vivaspin, 10 kDa MWCO, 4000 rcf, 15 min). Rh-sar-PD-trastuzumab was recovered in a 505 µL solution at a concentration of 5.338 mg/mL. Concentrations of antibody, and UV−Visible spectra were acquired on a Thermo Scientific NanoDrop 200c. Rh-sar-PD-trastuzumab was characterised by SDS-PAGE, UV-Vis and ESI-MS.

Rh-sar-PD-trastuzumab: SDS-PAGE analysis following conjugation showed two intense bands corresponding to fully re-bridged antibody (~145 kDa), and half-antibody (~75 kDa), as well as two lighter bands (heavy and light chain at 50 and 25 kDa, respectively). High resolution ESI-MS of Rh-sar-PD-trastuzumab, [HL + 2 Rh-tags] expected *m/z* = 73 773, observed *m/z* for = 73 782. HL = heavy chain + light chain (i.e. half antibody).

**Table S1.** $\lambda_{max}$ values for Rh-sar-PD-trastuzumab

| | $\lambda_{280}$ | $\lambda_{335}$ |
|---|---|---|
| **Rh-sar-PD-trastuzumab** | 0.958 | 0.172 |

UV-Vis spectroscopy was used to determine payload to antibody ratios. Given that $\varepsilon_{335}$ and $\varepsilon_{335}$ are known for simple dithiol-conjugated pyridazinedione motifs, a correction factor can be applied in the event that the conjugated payload has a competing absorption at $A_{280}$. This is the case for Rh-sar-PD-trastuzumab. In the following calculations:

- $\varepsilon_{280(\text{trastuzumab})}$ = 215,000

and

- $\varepsilon_{335(\text{pyridazinedione scaffolds})}$ = 9,100
- $\varepsilon_{280(\text{pyridazinedione scaffolds})}$ = 2,275
- Therefore, the correction factor:

$$\frac{2{,}275}{9{,}100} = 0.25$$

Calculating payload to antibody ratio:

$$c = \frac{A}{\varepsilon_{280}}$$

The derivations for calculating payload to antibody ratio from UV measurements are:

$$A_{280} = \left(\varepsilon_{280} + (0.25 \times n \times \varepsilon_{335})\right) \times c$$

and

$$A_{335} = n \times \varepsilon_{335} \times c$$

Thus:

$$n = \frac{\varepsilon_{280(trastuzumab)} \times A_{335}}{A_{280} \times \varepsilon_{335} + (0.25 \times A_{335} \times \varepsilon_{335})}$$

For Rh-sar-PD-trastuzumab, PAR = 4.06

*SDS-PAGE analysis of trastuzumab conjugates*

These methods are adapted from the literature.[5] A sample of immunoconjugate (3 µL) was diluted with water (10 µL), followed by addition of SDS-PAGE loading buffer (2 µL). This solution was mixed thoroughly and then heated to 65 °C for 5 min to denature the protein. The sample was then loaded onto the gel (12 % acrylamide resolving gel with 4% stacking gel), along with a molecular weight marker, a sample of trastuzumab (prepared in the same way), and a sample of reduced trastuzumab (also prepared in the same way). Separation of protein components was achieved by applying a constant voltage to the gels (35 mA, 40 min) in SDS running buffer. Gels were stained with Coomassie blue, destained, and then bright light imaged (Invitrogen, iBright FL1000, Thermo Fisher Scientific).

*ESI-MS analysis of bioconjugates*

Molecular masses were measured using an Agilent 6510 QTOF LC-MS system (Agilent, UK). An Agilent 1200 HPLC system was equipped with an Agilent PLRP-S, 1000A, 8 µM, 150 mm x 2.1 mm column. Bioconjugates were buffer exchanged to ammonium acetate (0.2 M, pH 6.9) using a Vivaspin (50 kDa MWCO) to a final concentration of 6.5 µM. To 100 µL of this bioconjugate solution was added PNGase F (1.0 µL, New England Biolabs) at 37 °C for 16 h. After this time, the sample was diluted to 1.8 µM with ammonium acetate buffer (0.2 M, pH 6.9). 10 µL of bioconjugate sample (1.8 µM) was injected on the column using mobile phase A (water-0.1% formic acid) and B (acetonitrile-0.1% formic acid) with an eluting gradient (as shown in Table S1) at a flow rate of 300 µl/min. The oven temperature was maintained at 60 °C. An Agilent 6510 QTOF mass spectrometer was operated in a positive polarity mode, coupled with an ESI ion source. The ion source parameters were set up with a VCap of 3500V, a gas temperature at 350 °C, a dry gas flow rate at 10 L/min and a nebulizer of 30 psig. MS ToF was acquired under conditions of a fragmentor at 350 V, a skimmer at 65 V and an acquisition rate at 0.5 spectra/s in a profile mode, within a scan range between 700 and 5000 m/z. The data was then analysed by deconvoluting to a zero charge mass spectrum using a maximum entropy deconvolution algorithm.

**Table S2.** LC-MS mobile phase gradient elution

| Time (min) | Solvent A (%) | Solvent B (%) |
|---|---|---|
| 0.0 | 85 | 15 |
| 1.0 | 85 | 15 |
| 1.50 | 68 | 32 |
| 2.0 | 68 | 32 |
| 7.0 | 50 | 50 |
| 9.0 | 5 | 95 |
| 10.0 | 5 | 95 |
| 12.0 | 85 | 15 |
| 15.0 | 85 | 15 |

*In vitro studies*

**LA-ICP-MS:** HCC1954 (HER2-positive) and MDA-MB-231 (HER2-negative) cells were cultured in RPMI medium, and then seeded onto separate Ibidi 12-well chamber slides (Gräfelfing Germany) in RPMI medium. A solution containing Rh-sar-PD-trastuzumab (0.5 µg in a total of 300 µL) was incubated with cells at 37 °C for 1 h in RPMI medium. In additional experiments, cells were co-incubated with excess trastuzumab (500 µg) to determine specificity of Rh-sar-PD-trastuzumab uptake. Following treatment with trastuzumab derivatives, media was removed, cells were washed with PBS, fixed with paraformaldehyde, dried, and analysed using LA-ICP-MS (Table S5).

**ICP-MS:** HCC1954 (HER2-positive) and MDA-MB-231 (HER2-negative) cells were cultured in RPMI medium. Cells were aspirated and washed with phosphate buffered saline (PBS), treated with trypsin to detach the cells from the flask, and washed again with RPMI media and then PBS. The cells were resuspended in Hank's

buffered saline solution (HBSS) with 0.2% bovine serum albumin (BSA), and aliquoted to give 1 × $10^6$ cells per tube. Solutions containing Rh-sar-PD-trastuzumab (1 µg in a total of 1 mL of RPMI) were incubated with the cells at 37 °C for 1 h. In additional experiments, cells were co-incubated with excess trastuzumab (100 µg) to determine specificity of Rh-sar-PD-trastuzumab uptake. Experiments were undertaken in technical quadruplicate. Cells were then washed and the cell pellet frozen at -80°C for ICP-MS analysis as described below.

*In vitro sample preparation for ICP-MS analysis*

Cell pellets were defrosted then oven dried at 65 °C for 1 h. After drying, 50 µL of Optima grade concentrated $HNO_3$ (67–69% w/w; Fisher Scientific) was added to the cell samples, which were left to digest in an oven at 70°C for 1 h. Reagent/antibody samples were also aliquoted (250 µL) and 50 µL of Optima grade concentrated $HNO_3$ was added to these samples and left to digest in an oven at 70 °C for 2 hrs. All sample were diluted by a factor of 25x using purified water with a resistivity ≥18.2 MΩ cm from an Milli Q system (Merk, Millipore). As part of the dilution, samples were doped with the internal standard Ga using a Teledyne Leeman Labs 100 mg/L Ga standard solution to obtain sample concentrations of 50 µg Ga/L. Calibration standards were made volumetrically using a 100 mg/L Sigma Aldrich TraceCert multi-element standard and a 0.5 M $HNO_3$ stock solution made from Optima grade concentrated $HNO_3$ and purified water. Element concentrations of the calibrants were between 0.01 and 100 µg/L and for all calibration solutions, and all calibration solutions, were doped with the same Teledyne Leeman Labs 100 mg/L Ga standard solution to obtain an internal standard concentration of 50 µg/L. For instrument details, see Table S4.

*In vivo ICP-MS uptake study and LA-ICP-MS imaging study*

All animal experiments were ethically reviewed by an Animal Welfare & Ethical Review Board at King's College London and carried out in accordance with the Animals (Scientific Procedures) Act 1986 UK Home Office regulations governing animal experimentation.

Eight to ten-week-old female NOD scid gamma (NSG) (NOD.Cg-Prkdc$^{scid}$ Il2rg$^{tm1Wjl}$/SzJ) mice (Charles River, Margate, UK) were subcutaneously inoculated with 1.5 x $10^6$ HCC1954 cells in 100 µL of 1:1 mixture of PBS/Matrigel (Corning™, #356234) in the left mammary fat pad between the 4$^{th}$ and 5$^{th}$ pairs of nipples. Experiments were conducted when the tumour volume reached ~100-150 $mm^3$. Tumour growth was measured using caliper measurements and the tumour volume was calculated using the formula: $V = (L*W^2)/2$ with V = volume, L = length (the long axis of the tumour), W = width (the short axis of the tumour).

HCC1954 tumour-bearing NSG mice were randomised into two groups. The first group (n of 4) was intravenously administered Rh-sar-PD-trastuzumab (500 µg) in saline. Mice were euthanised 72 h post-administration, followed by organ harvesting. The second group (n of 4) were intravenously administered trastuzumab (1 mg), followed 48 h later by Rh-sar-PD-trastuzumab (500 µg) in saline. Mice were euthanised 72 h post-administration of Rh-sar-PD-trastuzumab, followed by organ harvesting, weighing and tissue processing.

*Organ/tissue sample preparation for ex vivo imaging*

For LA-ICP-MS, harvested organs were placed in 4% PFA overnight, and then placed in PBS the following day.

The organs in PBS were dehydrated in: 70% EtOH (1 h), 95% EtOH 1 (1 h), 95% EtOH 2 (1 h), 100% EtOH 1 (1 h), 100% EtOH 2 (1 h), xylene 1 (45 min), xylene 2 (45 min), paraffin 1 (45 min) , paraffin 2 (overnight). Finally, the organs were paraffin embedded.

**LA-ICP-MS:** For the LA-ICP-MS experiments of Rh-sar-PD-trastuzumab distribution in tissue sections, slides were placed in the oven at 60°C for 1 h to deparaffinise sections. Tissue sections on each glass slide were then submerged into ultra-pure xylene (15 min) and ethanol (15 min), before being left to air-dry and ablated using LA-ICP-MS (Table S5).

**H&E staining:** Adjacent tissue sections to those prepared for LA-ICP-MS analysis were stained with haematoxylin and eosin (H&E) as follows. Firstly, paraffin-embedded slides were placed in the oven (60°C, 2 h). The slides were deparaffinised and hydrated through the following solutions: xylene 1 (5 min), xylene 2 (5 min), xylene 3 (5 min), 100 % EtOH wash (1 min), 100% EtOH 1 (5 min), 95% EtOH (5 min), 70% EtOH (5 min), dd$H_2O$ (5 min), haematoxylin Gill No.3 (8 min), dd$H_2O$ (10 min), acid alcohol solution (2 x dips), dd$H_2O$,

0.05% ammonia water solution (2 min), dd$H_2O$ (5 min), eosin (1 dip for 5 secs), 95% EtOH (rinse), 100% EtOH 2 (5 min), 100% EtOH 1 (5 min), xylene 3 (5 min), xylene 2 (5 min). Sections were mounted using DPX mounting medium, and imaged with a Hamamatsu Nanozoomer S630 digital slide scanner (UCL IQ path). Acid alcohol solution composition: 100% EtOH (210 mL), dd$H_2O$ (90 mL), concentrated HCl (3 mL). Ammonia water solution composition: $NH_4OH$ (250 µL) in ddH2O (500 mL).

**IHC staining and imaging:** Paraffin-embedded tissue sections were cut, dewaxed in xylene, and rehydrated through a graded ethanol series to water. Antigen retrieval was carried out by boiling the sections in Tris-EDTA buffer (10 mM Tris base, 1 mM EDTA, 0.05% Tween-20, pH 9) in a decloaking chamber at 90 °C. Endogenous peroxidase activity was blocked by incubating the sections in 3% $H_2O_2$ for 10 minutes at room temperature, followed by thorough rinsing in distilled water. Sections were then blocked in TBST containing 1% BSA and incubated overnight at 4 °C in a humidified chamber with primary rabbit monoclonal anti-HER2/ErbB2 (29D8) antibody (1:400 dilution; catalogue number 2165, Cell Signalling, UK). After washing in TBST, sections were incubated for 1 hour at room temperature with goat anti-rabbit HRP-conjugated secondary antibody (1:200 dilution; Thermo Fisher, UK) in blocking buffer. Signal was developed using a DAB peroxidase substrate kit (Vector Labs, UK). Slides were counterstained with haematoxylin, mounted with DPX, and scanned using a Hamamatsu NanoZoomer S20.

*Organ/tissue sample preparation for ICP-MS analysis*

The following organs/tissues/samples were collected for ICP-MS analysis: blood, heart, lungs, muscle (quadriceps), liver, spleen, kidneys, pancreas, stomach, small intestine, large intestine, bone (femur), skin (including fur), ovaries, uterine horn, and tumour. Due to the high mass of the liver, only a single lobe of the liver was sampled. The liver lobe and other fat-containing organs were cut into smaller sections to aid subsequent tissue processing and digestion.

The organs in PBS were dried at 70°C for 3 h and weighed in acid-cleaned HDPE centrifuge tubes. After obtaining the dry weight of each organ, 0.4 mL of Optima grade concentrated $HNO_3$ (68% *w/w*; Fisher Scientific trace metal grade acids) and 0.3 mL of Optima grade concentrated $H_2O_2$ (32% *w/w*; Fisher Scientific trace metal grade acids) were added to the tissue samples, followed by heating overnight in an oven at 70 °C to digest the samples. The digests were diluted by a factor of 20x with purified water with a resistivity ≥18.2 MΩ cm from a Milli-Q system (Merck Millipore) to achieve a final $HNO_3$ concentration of 0.8 M, and spiked with the internal 100 mg/L Ga standard to obtain a final concentration of 50 µg Ga/L. Element concentrations of the calibrants were between 0.01 and 100 µg/L and all calibration solutions, were doped with the same Teledyne Leeman Labs 100 mg/L Ga standard solution to obtain an internal standard concentration of 50 µg/L. For instrument details, see Table S4.

*ICP-MS measurements*

All concentration measurements were conducted on a Perkin Elmer NexION 350D Inductively Coupled Plasma Quadrupole Mass Spectrometer (ICP-MS) using helium gas in Kinetic Energy Discriminator mode. The introduction system to the instrument was a CETAC ASX-520 autosampler coupled to a SeaSpray glass nebuliser fitted to a quartz cyclonic spray chamber. Typical settings used for ICP-MS concentration measurements are presented in Table 1. The accuracy of concentration measurements was ensured through repeat measurements of acid blanks, calibration standards and CRM-TMDW-100 from High Purity Standards throughout the run. Data reduction was performed by normalising raw intensity measurements across the batch using gallium intensities and then background correcting the intensities by removing the batch-averaged intensity of blank measurements. The corrected isotopic intensity was converted to concentration measurements by external standardisation based on a six-point calibration curve.

*Elemental imaging by LA-ICP-MS*

In LA-ICP-MS experiments mapping the distribution of $^{103}$Rh in breast cancer cells, and tissue sections of mice administered Rh-sar-trastuzumab, an Iridia 193 nm ArF LA system (Teledyne Photon Machines, Bozeman) was used, equipped with the cobalt long-pulse ablation cell. The LA system was coupled to a Thermo Fisher Scientific iCAP TQ ICP mass spectrometer via the Aerosol Rapid Introduction System (ARIS). Full operational parameters for both the Iridia laser and iCAP TQ are provided in Table 2. Tuning of the instrument settings was performed using glass Standard Reference Material 612 from the National Institute for Standards and

Technology (NIST), optimising for low laser-induced elemental fractionation by monitoring $^{238}U/^{232}Th$, oxide formation rates (< 1%) via the $^{232}Th^{16}O^+/^{232}Th^+$ ratio, and the sensitivity of $^{59}Co$, $^{115}In$ and $^{238}U$. Element images of both HER2 cells and tissue sections were acquired in fixed dosage mode with a vertical and horizontal spatial resolution of 8 and 10 µm, respectively. To correct for instrumental drift, a series of NIST SRM 612 lines were ablated before and after each sample. Elemental data from the iCAP TQ and positional data from the Iridia were combined to generate elemental images using the HDF-based Image Processing software (HDIP, Teledyne Photon Machines). A bespoke in-house pipeline, written in Python, was used to analyse the reconstructed data and produce comparable elemental images. The pipeline consisted of removing negative values from instrument noise and replacing these values with zeros. A mask was then applied to each image to remove noise and reconstruction artefacts surrounding the samples. All data is presented on the same intensity scale for comparability (95% percentile). Final instrument parameters: element $^{103}Rh$; fluence 0.50 J/cm$^2$; dosage 15 n; rep rate 750 Hz; spot size 8 µm circle; washout 15 ms.

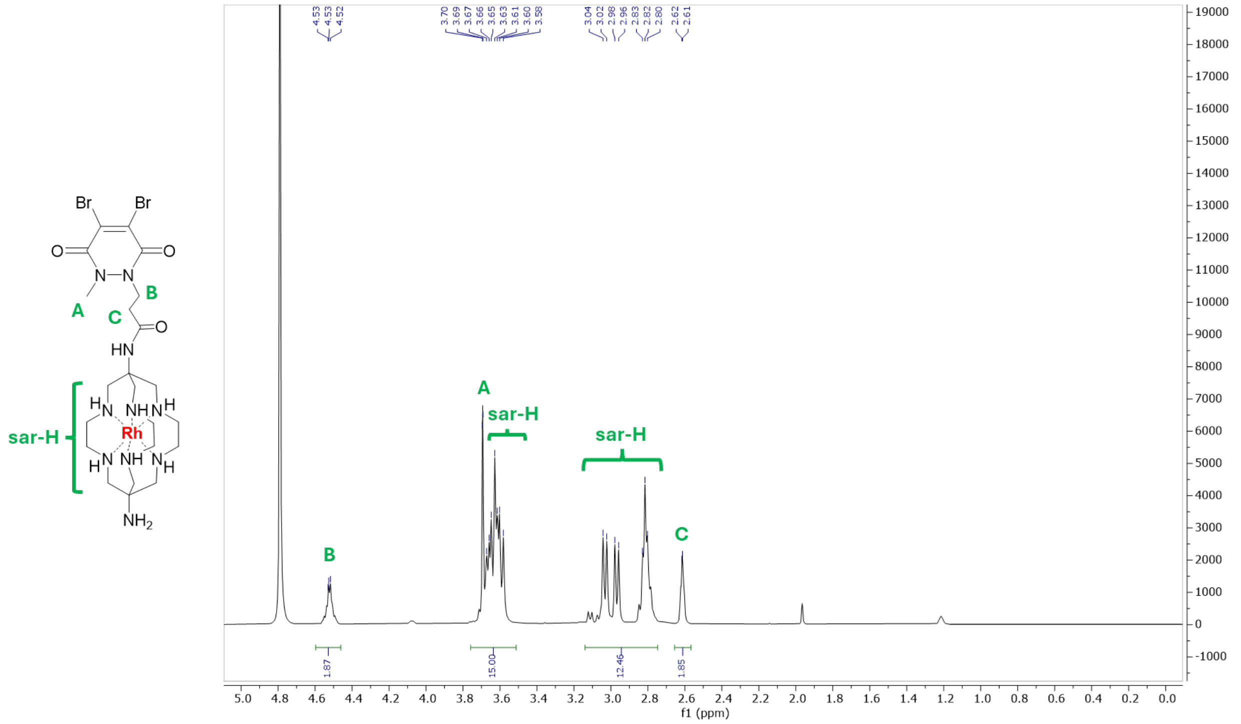


**Figure S1.** $^{1}H$ NMR spectra of Rh-sar-DBPD (700 MHz, $D_2O$).

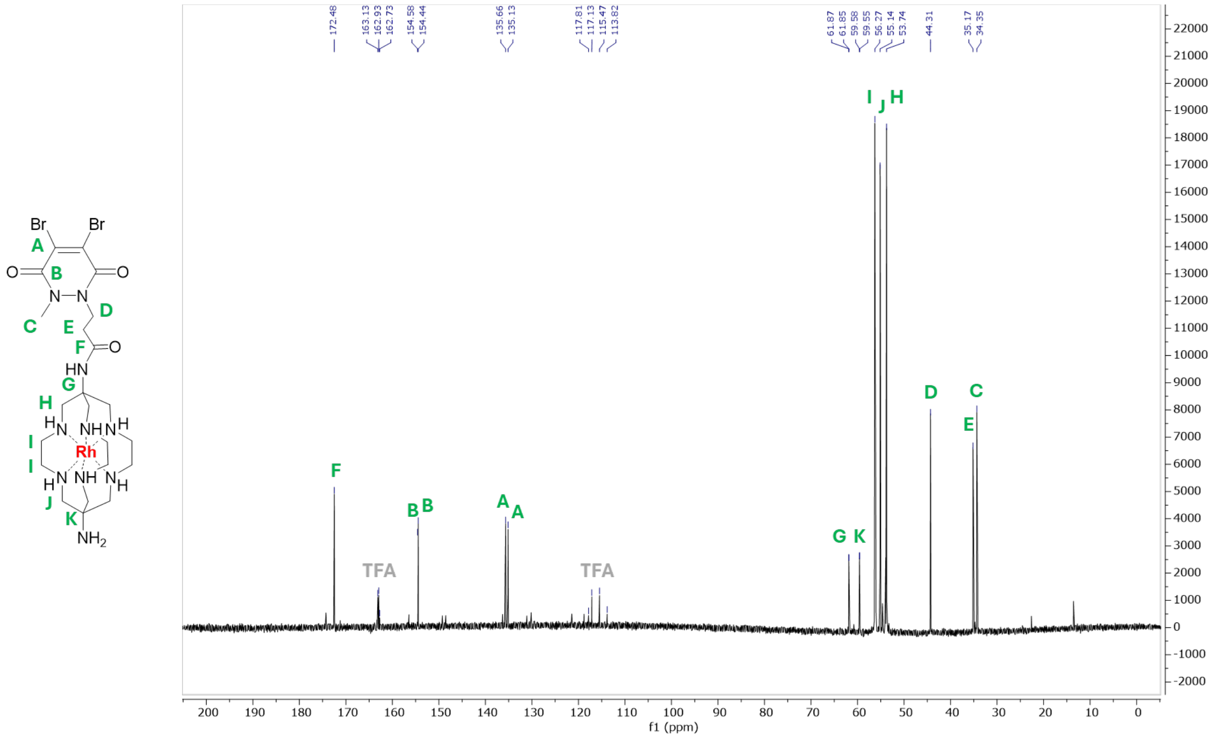


**Figure S2.** $^{13}C$ NMR spectra of Rh-sar-DBPD (175 MHz, $D_2O$).

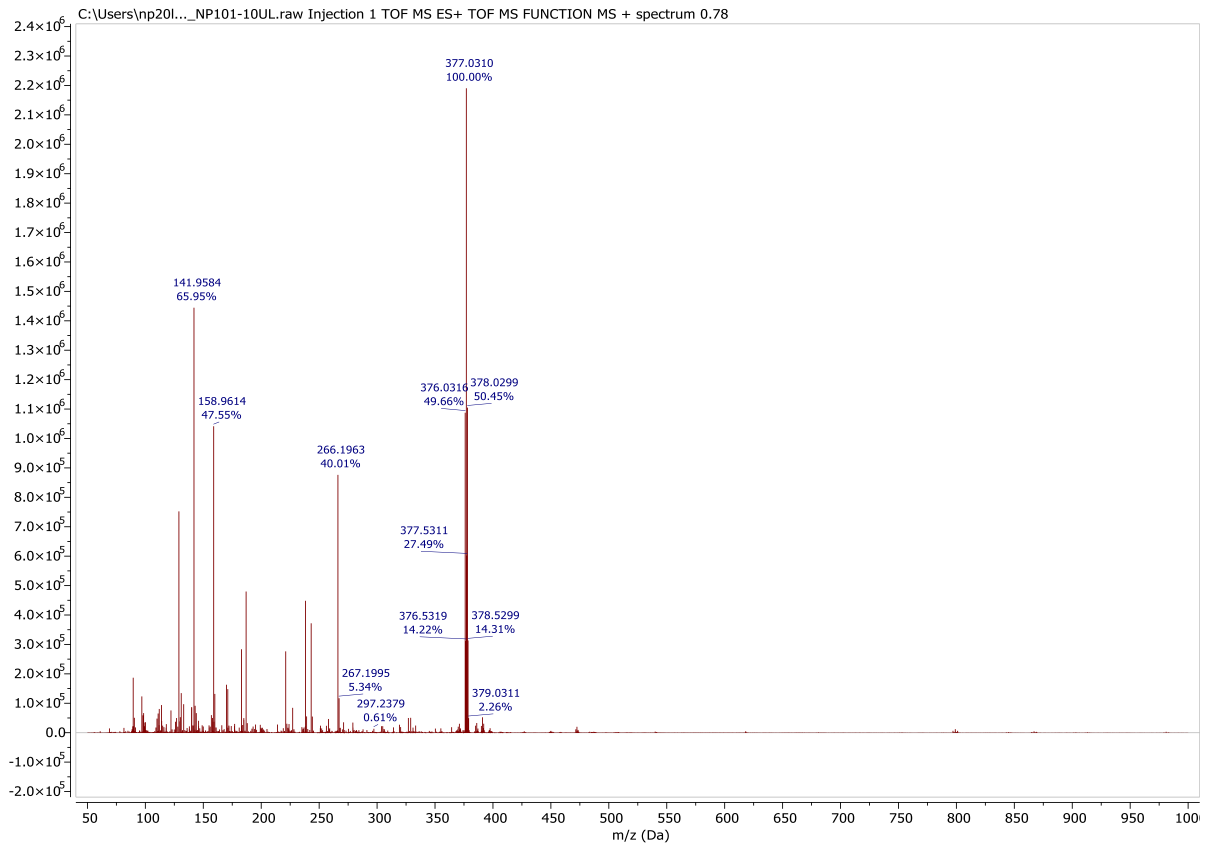


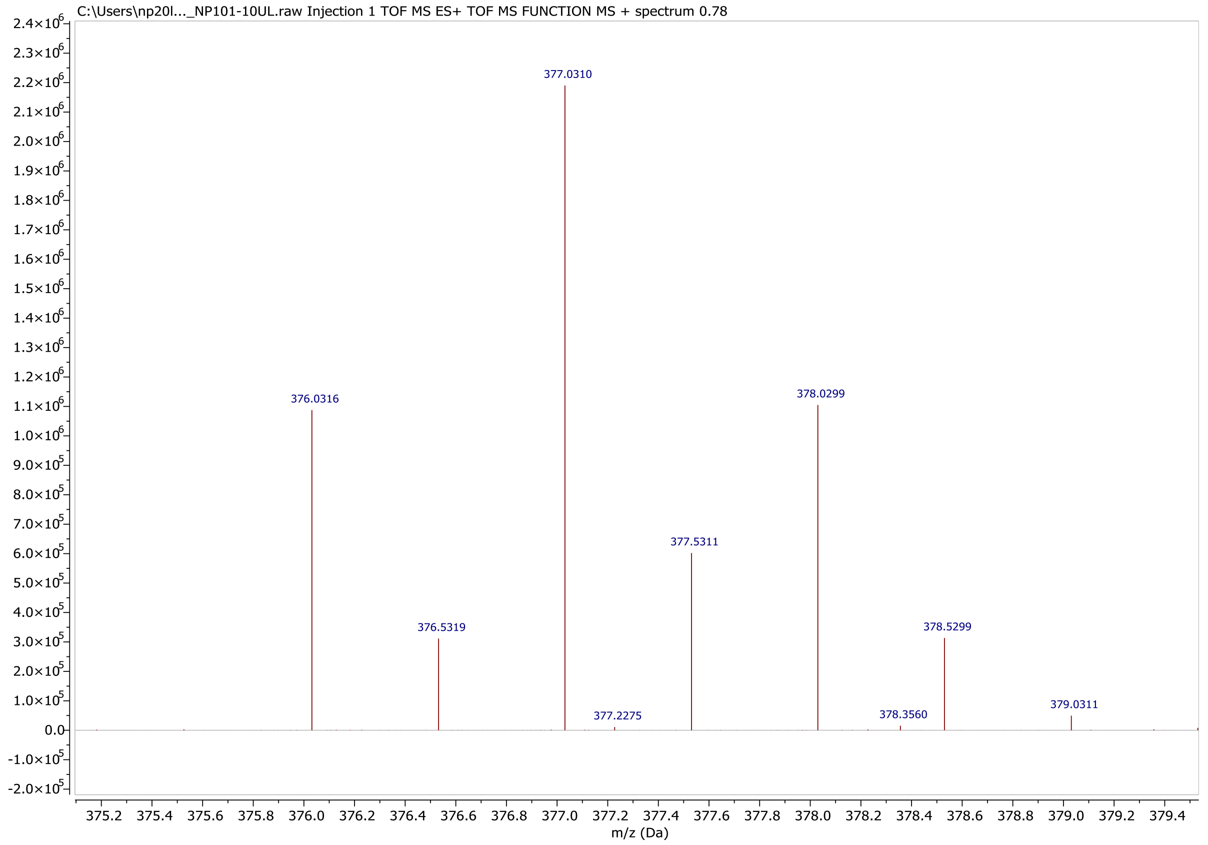


**Figure S3.** HR-ESI-MS of Rh-sar-DBPD (M = $[RhC_{22}H_{40}Br_2N_{10}O_3]^{3+}$). $[M - H]^{2+}$: observed = 377.0321, predicted = 377.0299.

**(a)** trastuzumab

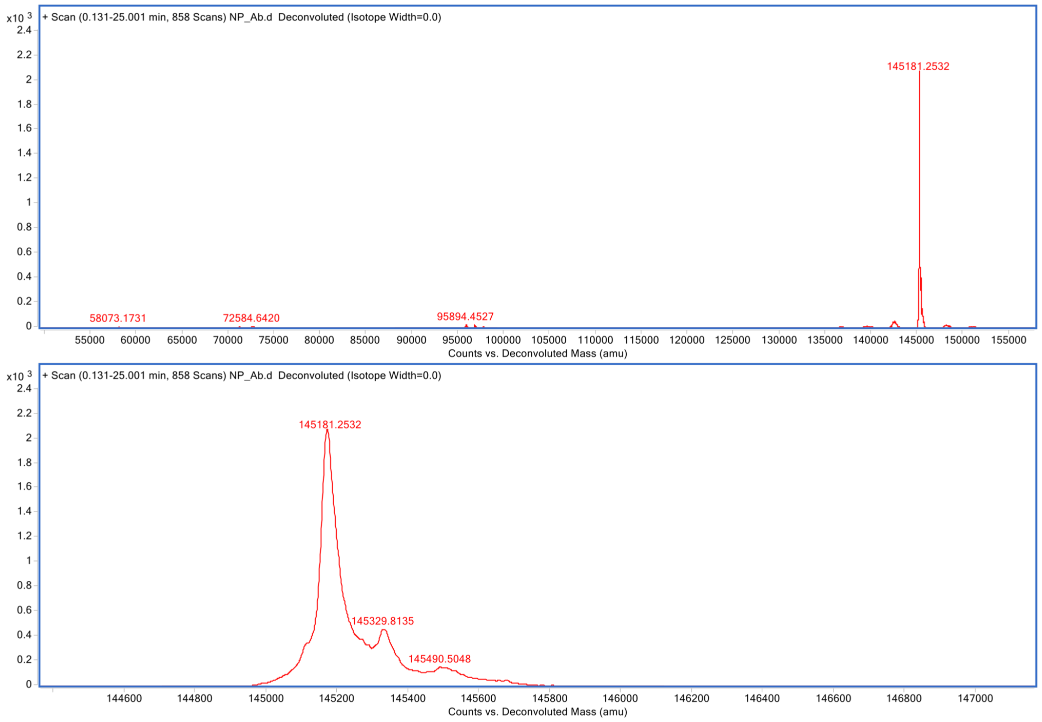


**(b)** Rh-sar-PD- trastuzumab

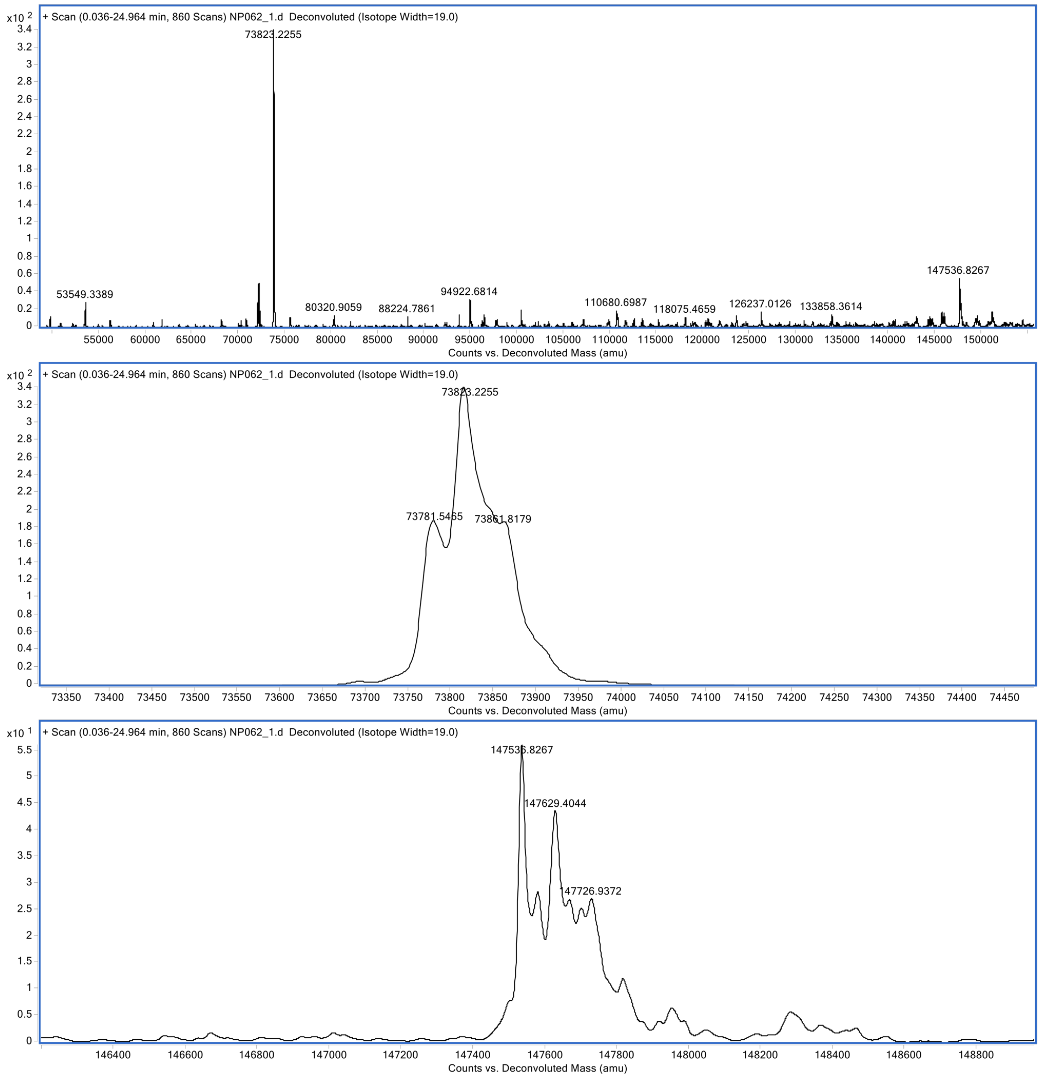


**Figure S4.** Deconvoluted HR-ESI-MS spectra of **(a)** trastuzumab and **(b)** Rh-sar-PD-trastuzumab.

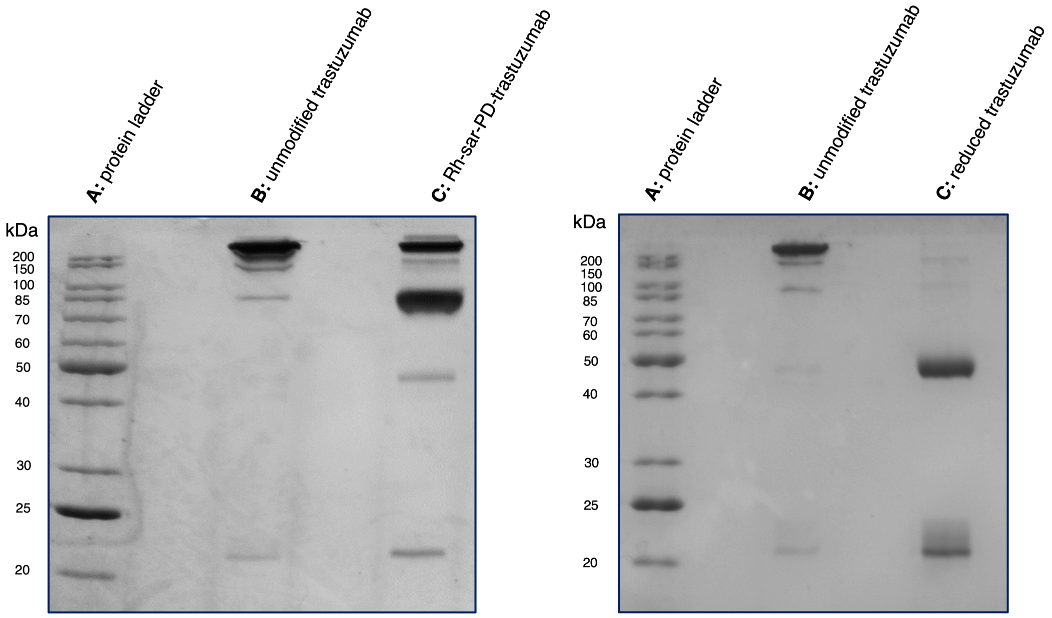


**Figure S5.** Full SDS-PAGE images of trastuzumab derivatives, including Rh-sar-PD-trastuzumab (left) and reduced trastuzumab (right).

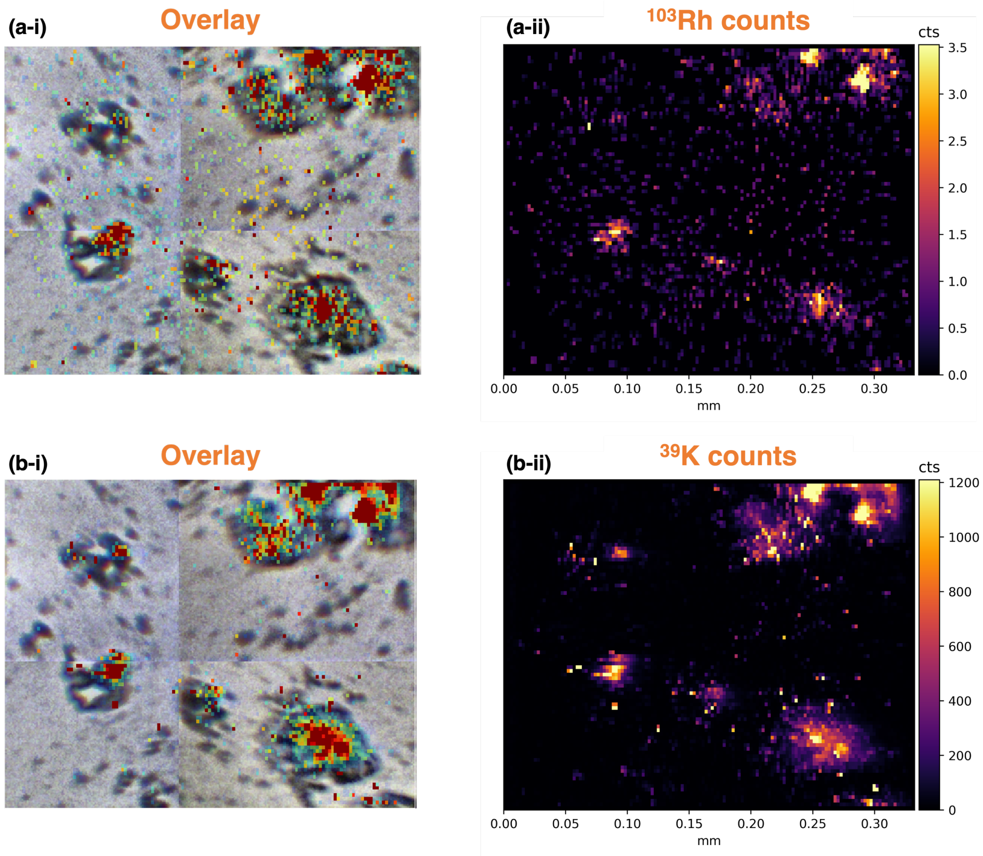


**Figure S6.** $^{39}$K is often used to delineate cells in LA-ICP-MS studies. Here, HER2-positive HCC1954 breast cancers cells were treated with Rh-sar-PD-trastuzumab, followed by LA-ICP-MS analysis using $^{103}$Rh and $^{39}$K channels (resolution = 3 μm). The spatial distribution of $^{39}$K signal correlated with $^{103}$Rh signal: **(a)** $^{103}$Rh channel, and **(b)** $^{39}$K channel, showing (i) the LA-ICP-MS image overlaid with the bright field image; (ii) the LA-ICP-MS image only.

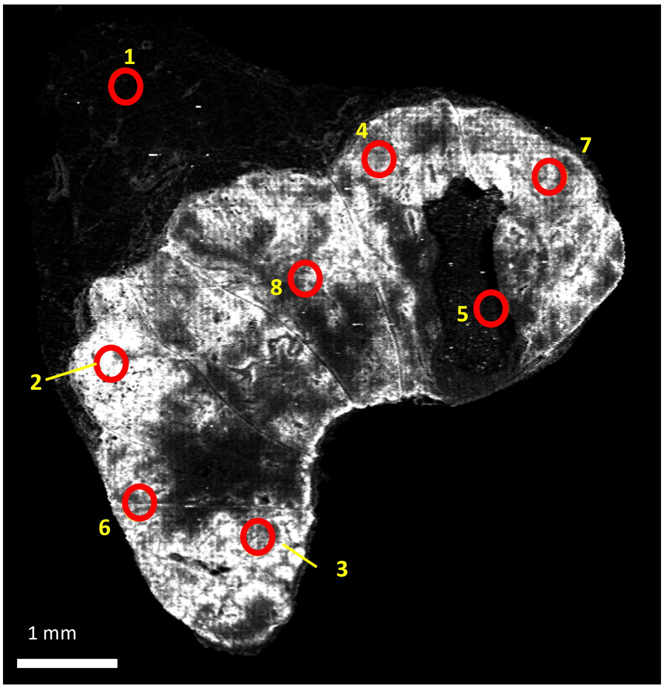


| Region | Average $^{103}$Rh counts per pixel |
|---|---|
| 1 | 1.89 |
| 2 | 100.03 |
| 3 | 40.73 |
| 4 | 44.41 |
| 5 | 4.21 |
| 6 | 45.19 |
| 7 | 46.05 |
| 8 | 39.94 |

**Figure S7.** $^{103}$Rh LA-ICP-MS image (resolution 10 μm) of a section of a tumour from a mouse administered Rh-sar-PD-trastuzumab. Image J was used to quantify regions of interest. Eight regions with the same area (830 mm$^2$) of interest were selected, to highlight heterogeneity of $^{103}$Rh uptake, which we postulate is a result of HER2 heterogeneity.

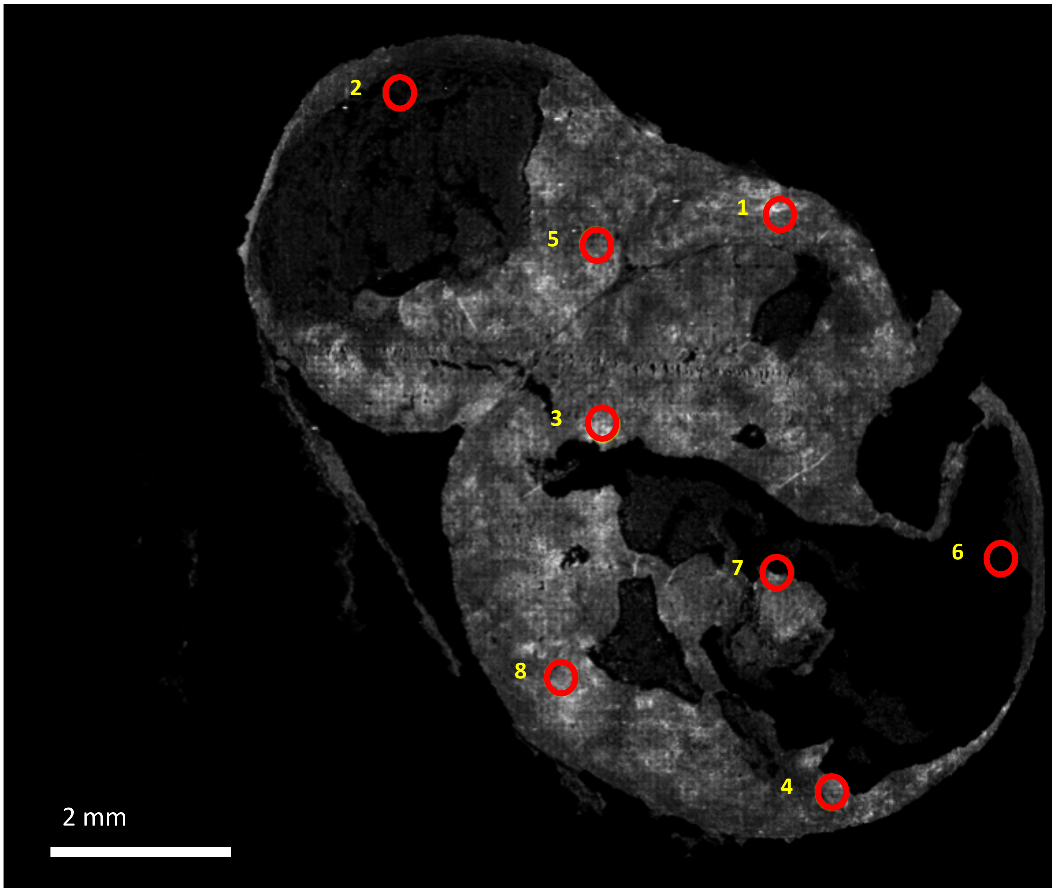


| Region | Average $^{103}$Rh counts per pixel |
|---|---|
| 1 | 23.56 |
| 2 | 2.07 |
| 3 | 29.53 |
| 4 | 17.69 |
| 5 | 17.46 |
| 6 | 0.19 |
| 7 | 8.80 |
| 8 | 24.05 |

**Figure S8.** $^{103}$Rh LA-ICP-MS image (resolution 10 μm) of a section of a tumour from a mouse administered native trastuzumab, followed by Rh-sar-PD-trastuzumab. Image J was used to quantify regions of interest. Eight regions with the same area (830 mm$^2$) of interest were selected, to highlight heterogeneity of $^{103}$Rh uptake, which we postulate is a result of HER2 heterogeneity.

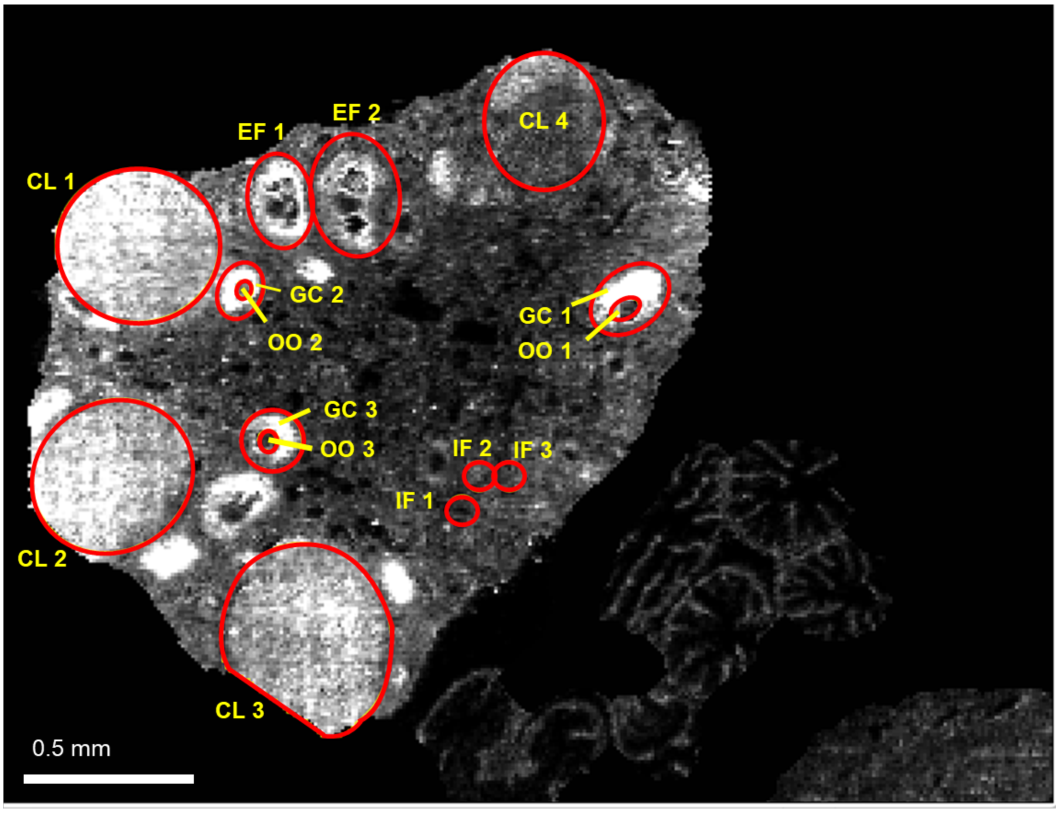


| Structure | Abbreviation | Average $^{103}$Rh counts per pixel |
|---|---|---|
| Corpus Luteum 1 | CL 1 | 41.339 |
| Corpus Luteum 2 | CL 2 | 37.519 |
| Corpus Luteum 3 | CL 3 | 32.167 |
| Corpus Luteum 4 | CL 4 | 17.295 |
| Granulosa Cells 1 | GC 1 | 47.755 |
| Granulosa Cells 2 | GC 2 | 49.726 |
| Granulosa Cells 3 | GC 3 | 31.491 |
| Oocyte 1 | OO 1 | 17.777 |
| Oocyte 2 | OO 2 | 27.176 |
| Oocyte 3 | OO 3 | 11.965 |
| Empty Follicle 1 | EF 1 | 33.157 |
| Empty Follicle 2 | EF 2 | 22.389 |
| Immature Follicle 1 | IF 1 | 8.964 |
| Immature Follicle 2 | IF 2 | 13.190 |
| Immature Follicle 3 | IF 3 | 12.911 |

**Figure S9.** $^{103}$Rh LA-ICP-MS image (resolution 10 μm) of a section of an ovary from a mouse administered Rh-sar-PD-trastuzumab. Image J was used to quantify regions of interest. A contiguous Haematoxylin & Eosin stained section was used to define regions of interest (Figure 6, main manuscript). The table shows the average counts per pixel for each region of interest.

(a) HER2_Nuclei (Haematoxylin)

(b) Nuclei (Haematoxylin)

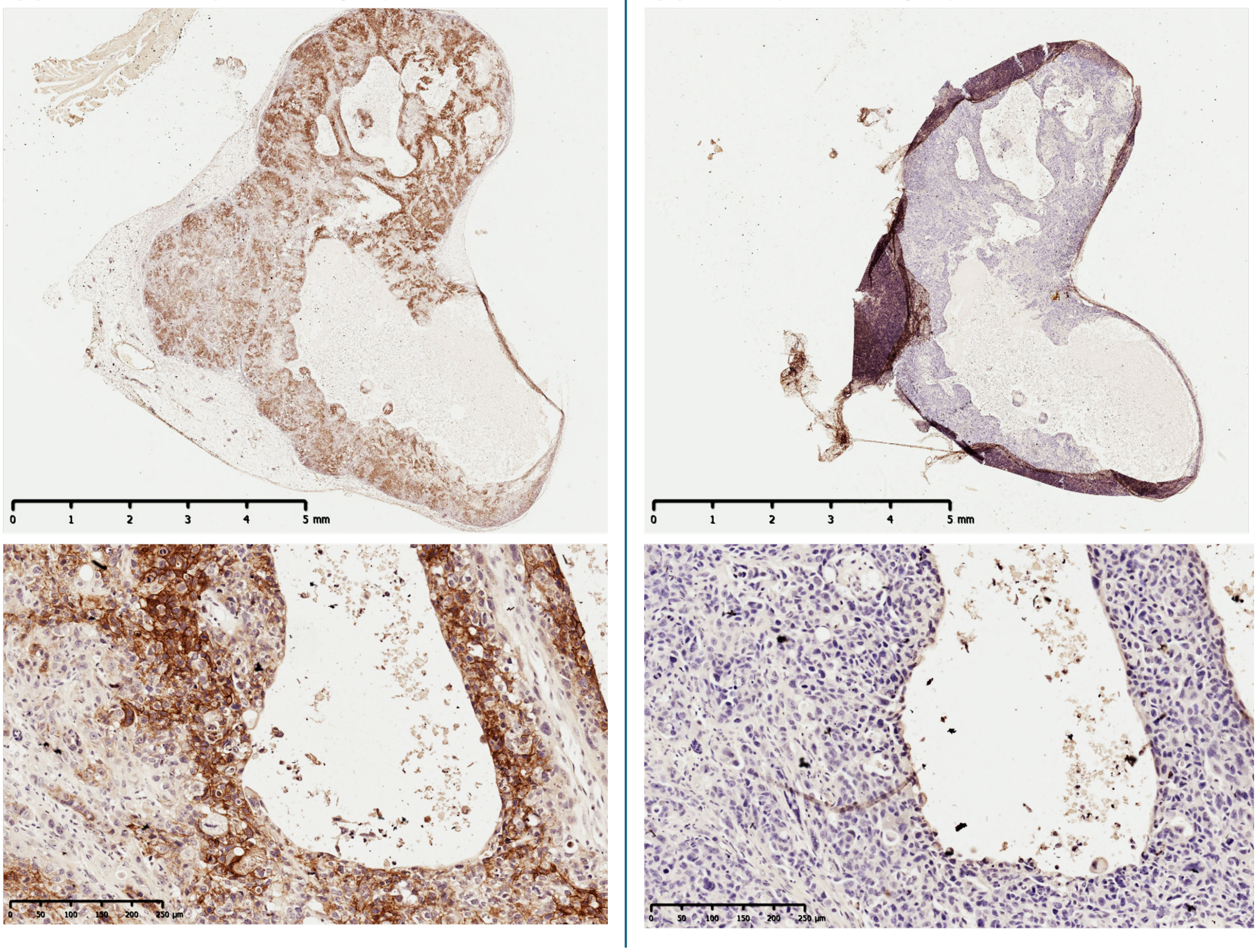


**Figure S10.** HER2 immunohistochemistry staining: (a) A HCC1954 breast cancer tumour section (from an NSG mouse bearing an orthotopic human HCC1954 breast cancer tumour) was incubated with primary anti-HER2 antibody, followed by detection using an HRP-conjugated secondary antibody and DAB as the chromogenic substrate. Slides were counterstained with haematoxylin; (b) A contiguous section was incubated with blocking buffer, followed by detection using an HRP-conjugated secondary antibody and DAB as the chromogenic substrate. Slides were counterstained with haematoxylin.

**Table S3.** Comparison of experimental conditions for reaction of Rh-sar-DBPD with trastuzumab

| **Method** | **Payload to antibody ratio** (determined by UV-Vis spectroscopy) |
|---|---|
| **Method 1:** Rh-sar-DBPD was mixed with trastuzamab for 1 h at 4°C, followed by addition of TCEP. The solution was left to react at 4 °C for 16 h. This method duplicates previously reported approaches.[3] | 3.33 |
| **Method 2:** Rh-sar-DBPD and TCEP were simultaneously added trastuzamab at 25 °C, and left to react for 4 h. | 3.44 |
| **Method 3:** TCEP was added to trastuzamab, and left to react at 37 °C for 1.5 h. Subsequently, TCEP was removed by ultrafiltration, and Rh-sar-DBPD was added to reduced trastuzamab and incubated for 3 h at 37 °C. This method duplicates previously reported approaches.[6] | 4.06 |

**Table S4.** Experimental data acquisition parameters used for ICP-MS

| | |
|---|---|
| **Perkin Elmer NexION 350D ICP-MS** | |
| RF power | 1600 W |
| Ar plasma gas flow rate | 18 L $min^{-1}$ |
| Ar auxiliary gas flow rate | 1.2 L $min^{-1}$ |
| Nebuliser gas flow | ~0.96 L $min^{-1}$ |
| CRC operation mode | KED |
| Helium KED gas flow | 4.5 mL $min^{-1}$ |
| **Method acquisition** | |
| Scanning mode | Peak-hopping |
| Dwell time | 100 ms $amu^{-1}$ |
| Sweeps per reading | 1 |
| Readings per replicate | 5 |
| RPa | 0 |
| RPq | 0.25 |
| **Sample introduction** | |
| Sample uptake rate | ~300 μL $min^{-1}$ |
| Sample flush | 75 s |
| Read delay | 20 s |
| Rinse time | 100 s |

**Table S5.** Experimental data acquisition parameters used for LA-ICP-MS imaging

| | **Tissue** | **Breast cancer cells** | **NIST SRM 612** |
|---|---|---|---|
| **Teledyne Photon Machines Iridia** | | | |
| Energy density (J $cm^{-2}$) | 0.5 | 0.5 | 3 |
| Repetition rate (Hz) | 500 | 750 | 100 |
| Scan speed (µm $s^{-1}$) | | | |
| Beam waist diameter (µm) | 10 (square) | 8 (circle) | 35 (square) |
| Scanning Mode | Fixed Dosage | Fixed Dosage | Fixed Dosage |
| Scanning Direction | Uni-directional | Uni-directional | Uni-directional |
| Effective Dosage | 10 | 15 | 15 |
| Helium carrier gas flow rate (L $min^{-1}$) | 0.3 | 0.3 | 0.3 |
| Washout (ms) | 15 | 15 | 15 |
| **Thermo Fisher Scientific ICAP TQ** | | | |
| RF power (W) | 1500 | 1500 | 1500 |
| Ar plasma gas flow rate (L $min^{-1}$) | 14 | 14 | 14 |
| Ar auxiliary gas flow rate (L $min^{-1}$) | 0.8 | 0.8 | 0.8 |
| Nebuliser gas flow rate (L $min^{-1}$) | 1.03 | 1.03 | 1.03 |
| CR gas flow rate (L $min^{-1}$) | 0.16 ($O_2$) | 0.16 ($O_2$) | 0.16 ($O_2$) |
| ICP-MS mode | TQ | TQ | TQ |
| Acquired m/z ratios (amu) | $^{103}$Rh | $^{103}$Rh | $^{103}$Rh |
| Respective dwell times (ms) | 20 | 15 | 15/20 |
| Total scan cycle time (ms) | 20 | 15 | 15/20 |

**Table S6.** *Ex vivo* biodistribution of female NSG mice bearing orthotopic HCC1954 tumours ($n$ = 4), administered either Rh-sar-PD-trastuzumab (500 μg) or Rh-sar-PD-trastuzumab (500 μg) with excess trastuzumab (1 mg) to block HER2 receptors. Quantification is based on $^{103}$Rh ICP-MS measurements of tissue digests.

| Organ | Rh-sar-PD-trastuzumab | Rh-sar-PD-trastuzumab + block | *P* value |
|---|---|---|---|
| Blood | 0.50±0.15 | 0.21±0.04 | 0.024 |
| Heart | 0.34±0.06 | 0.23±0.04 | 0.031 |
| Lungs | 0.27±0.12 | 0.26±0.10 | 0.878 |
| Muscle | 0.06±0.02 | 0.05±0.01 | 0.365 |
| Liver | 3.36±0.70 | 3.00±0.84 | 0.585 |
| Spleen | 1.88±0.40 | 2.23±0.52 | 0.328 |
| Kidneys | 1.67±0.24 | 1.35±0.25 | 0.111 |
| Pancreas | 0.13±0.03 | 0.09±0.02 | 0.0538 |
| Stomach | 0.50±0.08 | 0.20±0.04 | 0.002 |
| Small intestine | 0.61±0.20 | 0.50±0.13 | 0.387 |
| Large intestine | 0.49±0.13 | 0.19±0.07 | 0.011 |
| Bone | 0.21±0.07 | 0.18±0.05 | 0.542 |
| Skin + fur | 0.16±0.07 | 0.21±0.05 | 0.278 |
| Ovaries | 0.48±0.07 | 0.20±0.09 | 0.003 |
| Uterine horn | 0.52±0.08 | 0.41±0.06 | 0.066 |
| Tumour | 1.67±0.21 | 0.48±0.19 | 0.00018 |

## References


1 R. J. Geue, T. W. Hambley, J. M. Harrowfield, A. M. Sargeson and M. R. Snow, *J. Am. Chem. Soc.*, 1984, **106**, 5478–5488.

2 J. M. B. Harrowfield, A. J. Herlt, P. A. Lay, A. M. Sargeson, A. M. Bond, W. A. Mulac and J. C. Sullivan, *J. Am. Chem. Soc.*, 1983, **105**, 5503–5505.

3 C. Bahou, D. A. Richards, A. Maruani, E. A. Love, F. Javaid, S. Caddick, J. R. Baker and V. Chudasama, *Org. Biomol. Chem.*, 2018, **16**, 1359–1366.

4 M. Morais, J. P. M. Nunes, K. Karu, N. Forte, I. Benni, M. E. B. Smith, S. Caddick, V. Chudasama and J. R. Baker, *Org. Biomol. Chem.*, 2017, **15**, 2947–2952.

5 M. Morais, N. Forte, V. Chudasama and J. R. Baker, in *Methods in Molecular Biology*, Humana Press Inc., 2019, vol. 2033, pp. 15–24.

6 C. Bahou and V. Chudasama, *Org. Biomol. Chem.*, 2022, **20**, 5879–5890.
